# Soil factors enhance amino acid and peptide accumulation in tuberous roots of *Pseudostellaria heterophylla*

**DOI:** 10.3389/fpls.2025.1728201

**Published:** 2025-12-03

**Authors:** Huiyong Zheng, Hong Chen, Meixia Zheng, Yuqing Niu, Jiajia Zhang, Wenbao Luo, Yujing Zhu, Hailan Su, Yanming Zhu

**Affiliations:** 1 Institute of Digital Agriculture, Fujian Academy of Agricultural Sciences, Fuzhou, Fujian, China; 2 Institute of Crop Sciences, Fujian Academy of Agricultural Sciences (Fujian Germplasm Resources Center), Fuzhou, China; 3 Institute of Environmental Microbiology, College of Resources and Environment, Fujian Agriculture and Forestry University, Fuzhou, China; 4 College of Horticulture, Fujian Agriculture and Forestry University, Fuzhou, China

**Keywords:** *Pseudostellaria heterophylla*, rhizosphere soil, bacterial community structure, metabolome, amino acids and peptides

## Abstract

**Introduction:**

*Pseudostellaria heterophylla* (PSH) is a renowned medicinal and culinary plant. However, soil-related factors limiting in the improvement of its yield and quality in practice remain poorly understood.

**Methods:**

We sampled two PSH varieties and their associated rhizosphere soils from two sites in Ningde City, Fujian Province, China, including Chouling village (Z) and Wuyang village (W), to analyze the basic soil physicochemical properties, soil bacterial communities, and untargeted metabolomics of both soils and tuberous roots samples.

**Results:**

Our results showed that the rhizosphere soil at site Z had significantly higher concentrations of calcium (Ca), magnesium (Mg), manganese (Mn), available phosphorus (AP), and available potassium (AK), but a lower concentration of alkaline hydrolyzable nitrogen (AN). Soil AN and AK were identified as key determinants of bacterial community structure, showing negative and positive correlation with numerous microbial phyla and genera, respectively. Site Z exhibited higher abundances of functional bacterial phyla, including Desulfobacterota, Nitrospirota, and Elusimicrobiota, as well as dominant genera of Acidobacteriota. Amino acids and peptides (AAs) were the most abundant class of differential metabolites (DMs) in both the rhizosphere soil and tuberous roots of PSH. The accumulation of AAs in tuberous roots was positively correlated with soil pH, electrical conductivity (EC), and potassium (K) levels, but negatively correlated with AN. Furthermore, specific microbial taxa and soil DMs at site Z were positively associated with AA abundance in the tuberous roots.

**Discussion:**

These findings suggest that appropriate soil nitrogen levels coupled with relatively high potassium availability, pH, and EC conditions are conducive to AAs accumulation in PSH tuberous roots.

## 1 Introduction

*Pseudostellaria heterophylla* (PSH), a member of the Caryophyllaceae family, is a renowned medicinal and culinary plant (Chinese Pharmacopoeia Commission, 2020). It is valued for its anti-aging properties and efficacy in treating spleen deficiency, cough, anorexia, hypoglycemia, hyperlipidemia, and heart palpitations (Qin et al., 2025). The tuberous roots of PSH are rich in bioactive compounds, such as cyclic peptides, amino acids (AAs), peptides, and their conjugates (Sha et al., 2023). The quality of PSH is influenced by both genetic factors and the ecological environment (Li et al., 2024; Shi et al., 2025). For instance, Sha et al. (2023) reported variations in the accumulation of heterophyllin B (an active cyclic peptide) in PSH samples from different provinces in China, including Guizhou, Fujian, Jiangsu, Anhui, Hebei, Shandong, and Shanxi. Similarly, Hua et al. (2016a, 2016b) identified distinct metabolic profiles in PSH samples from Jurong and Zherong, indicating regional influences on metabolite composition. Ecological and environmental factors have been highlighted as predominant controllers of protein expression related to AA synthesis in PSH (Hua et al., 2019).

Soil properties play a role in determining plant quality. Zheng et al. (2024) reported that soil pH negatively affects water-soluble extracts in PSH, while soil organic carbon and available nitrogen (N) have positive effects. The application of phosphorus-modified biochar was shown to increase polysaccharide (by 2.9%) and saponin (by 78.8%) contents in PSH tuberous roots (Ng et al., 2022). The bacterial communities in the rhizosphere are highly specific and vary across different habitats (Korenblum et al., 2022). These microorganisms can influence plant metabolite synthesis by modulating hormone secretion and host immune responses, thereby enhancing plant quality (Korenblum et al., 2022). Conversely, metabolites released by soil microbes can elicit plant responses, that feedback on microbial community composition (Qiao et al., 2024). For example, Lin et al. (2024) demonstrated that a fertilizer containing *Bacillus* and *Burkholderia* strains improved soil structure, promoted eutrophic bacterial communities, and increased saponin and polysaccharide contents in PSH. Similarly, Wu et al. (2021) observed increased cyclic peptide content in PSH following the application of a bacterial fertilizer containing *Pseudomonas* and *Bacillus*. Plants can convert amino acids into specialized metabolites that serve as signaling molecules or shape the microbiome to their advantage (Lanfranco et al., 2018). Plant amino acid transporters may be targeted by microbes to enhance nutrient availability, and are regulated by the plant immune system to differentially support beneficial microbes or restrict pathogens (Moormann et al., 2022). However, current research has primarily focused on using microbial fertilizers to mitigate continuous cropping challenges, leaving their roles in quality improvement inadequately explored.

Fujian Province is a primary cultivation region for PSH in China. Zherong *P. heterophylla*, in particular, was recognized as a National Geographical Indication Product in 2011 (Wu et al., 2019). We hypothesize that the rhizosphere microenvironment in Zherong is more conducive to the accumulation of secondary metabolites and overall quality of PSH. To test this, we sampled two PSH varieties (Zheshen 1 and Zheshen 4) and their rhizosphere soils from Chouling village (Z) and Wuyang village (W) in Fujian Province. Our objectives were to analyze: (1) the physicochemical properties and bacterial community structure in the rhizosphere soils, and (2) the metabolite profiles in both the rhizosphere soils and tuberous roots. This study aims to identify the main soil factors that improve the medicinal properties of PSH, with a particular focus on the accumulation of AAs and peptides. Our findings are expected to advance the understanding of soil factors controlling PSH quality and provide a theoretical basis for developing cultivation techniques to produce high-quality PSH.

## 2 Materials and methods

### 2.1 Sampling sites

Samples were collected from two sampling sites in Ningde city, Fujian Province, China: Chouling village (119.36°E, 26.13°N; designated Z) in Zherong County, and Wuyang village (119.98°E, 26.99°N; designated W) in Fuding County. Both regions experience a typical subtropical monsoon climate. The preceding crop in both plots was the same PSH variety. Two PSH varieties, Zheshen 1 (T1) and Zheshen 4 (T4), were sampled at both sites. T4 is a hybrid variety derived from T1 as the maternal parent. Field management practices were consistent across the experimental period. Sampling was conducted in July 2023, after the aboveground parts had senesced. Four sample types were collected: seedlings and rhizosphere soil of variety T1 from site Z (Z1), T4 from site Z (Z4), T1 from site W (W1), and T4 from site W (W4).

### 2.2 Sample collection

Plants with intact root systems were carefully excavated. The soil closely adhering to the surface of tuberous roots (within approximately 2 mm) was defined as rhizosphere soil. The topsoil was removed with a sterilized shovel, and the root systems were carefully excavated. After gently shaking off loosely attached soil, the rhizosphere soil was collected by brushing the root surface with a separate sterile brush. At each site, nine seedlings per variety (18 seedlings total per site) were sampled. For each variety, three seedlings and their associated rhizosphere soils were pooled to form one biological replicate (including three technical replicates).

Soil samples were placed in sterile centrifuge tubes, transported on dry ice to the laboratory, and stored at -80 °C. A portion of each fresh rhizosphere soil sample was sent to Shanghai Majorbio Biotechnology Company for microbial community and metabolomic analyses. The tuberous roots were washed with tap water, followed by deionized water, flash-frozen in liquid N_2_ and stored at -80 °C before being sent for non-targeted metabolomic analysis.

### 2.3 Determination of soil physicochemical properties

The remaining soil samples were used to determine physicochemical properties. Soil pH and electrical conductivity (EC) were measured in a 1:5 (w/v) soil-to-water extract using a pH meter and conductivity meter, respectively. Soil organic matter (SOM) content was quantified by the potassium dichromate oxidation method with external heating. Total nitrogen (TN) was determined by the Kjeldahl method, and alkaline hydrolyzable nitrogen (AN) by the alkaline diffusion method. Total phosphorus (TP) was measured by the molybdenum-antimony colorimetric method, and available phosphorus (AP) was determined by the NaHCO_3_ extraction method. Available potassium (AK) was evaluated via the ammonium acetate extraction method followed by flame photometry. Detailed methodologies are described by Bao (2000).

### 2.4 Digestion of soil samples and determination of element concentrations

Soil samples were digested using an electric heating digester (Labtech, ED54) following the protocol of Liao et al. (2016). Briefly, 1 g of soil was weighed into a 100 mL Erlenmeyer flask, mixed with 10 mL of HNO_3_:HClO_4_ (3:2, v/v)mixture, covered with a small funnel, and left to stand overnight. The following day, the mixture was digested at 160 °C until the solution became colorless, and the soil residue appeared off-white. Digestion continued until white fumes appeared. The flask was cooled for 1–2 min, then 10 mL of HCl (1:1, v/v) was added, and the flask heated in a boiling water bath for 10 min. After cooling to room temperature, the solution was transferred to a 50 mL volumetric flask. Concentrations of calcium (Ca), zinc (Zn), iron (Fe), manganese (Mn), magnesium (Mg) and copper (Cu) in the digestate were analyzed by ICP–MS (Thermo Fisher, iCAP Qc). A standard reference material (shrub leaves, GBW07603, GSV-2) from the National Research Center for Certified Reference Materials of China was used for quality control, with recoveries ranging from 95% to 108%.

### 2.5 Analysis of the soil microbial community structure

Soil microbial community analysis followed Zhang et al. (2025). DNA was extracted using the TGuide S96 Magnetic Soil/Stool DNA Kit (Tiangen Biotech Co., Ltd., Beijing, China) according to the manufacturer’s instructions. DNA concentration was measured using a Qubit dsDNA High Sensitivity Assay Kit on a Qubit 4.0 Fluorometer (Invitrogen, Thermo Fisher Scientific, USA). The V3–V4 region of bacterial 16S rRNA gene was amplified with primers 338F (5’-ACTCCTACGGGAGGCAGCAG-3’) and 806R (5’-GGACTACHYGGGTWTCTAAT-3’). PCR amplification involved 25 cycles using KOD One PCR Master Mix (Toyobo Life Science). Amplicons were purified with Agencourt AMPure XP Beads (Beckman Coulter, USA), quantified as above, and sequenced on a PacBio Sequel II 8 M SMRT cell using the Sequel II Sequencing Kit (v 2.0). Bioinformatics analysis was performed on the Majorbio Cloud Platform (Majorbio Bio-Pharm Technology Co., Ltd., Shanghai, China).

### 2.6 Untargeted metabolomics analysis

Untargeted metabolomics analysis of soil and tuberous root samples followed Zhu et al. (2022). Approximately 50 mg of sample was extracted with 400 µL of methanol:water (4:1, v/v) containing 0.02 mg·mL^–1^ L-2-chlorophenylalanine (internal standard). The mixture was homogenized at -10 °C using a high-throughput tissue crusher (Wonbio-96c, Shanghai Wanbo Biotechnology Co., Ltd.) at 50 Hz for 6 min, sonicated (40 kHz, 30 min, 5 °C) and incubated at -20 °C for a 30 min to precipitate proteins. After centrifugation (13,000 × g, 15 min, 4 °C), the supernatant was subjected to LC–MS/MS analysis. Metabolite separation was performed on a Thermo UHPLC system with an ACQUITY UPLC HSS T3 column (100.0 mm × 2.1 mm, 1.8 µm; Waters, USA). Mass spectrometric data were acquired using a Thermo UHPLC-Q Exactive HF-X mass spectrometer with an electrospray ionization source in data-dependent acquisition mode. Raw data were processed using Progenesis QI (v 2.3, Nonlinear Dynamics, Waters, USA) for peak detection and alignment.

### 2.7 Data processing and statistical analysis

Soil physicochemical data were processed using Excel 2019. Statistical analysis was performed with SPSS (SPSS Inc., USA), and graphs were generated with Origin (v 2024b). One-way and two-way ANOVA were employed to assess the significant differences between sites and varieties (*p* < 0.05). Data normality was verified using the Shapiro-Wilk test (*p* > 0.05); only normally distributed data were subjected to ANOVA (Zhu et al., 2020). Metabolomics data were analyzed on the Majorbio Cloud Platform using R Software (v3.1.1, picante, v1.8.2) for alpha diversity, QIIME 1.8.0 for principal coordinate analysis (PCoA), and Python 3.x (scipy v0.17.1) for ANOVA with the Tukey−Kramer *post hoc* test and Spearman’s correlation analysis (Zhu et al., 2025). The variable importance in projection (VIP) was calculated from the OPLS-DA model. *P*_values were derived from paired Student’s t tests. Metabolites with VIP > 1 and *p* < 0.05 were considered significant (Zhu et al., 2022). The Mantel test was utilized to examine the relationships between soil factors and bacterial communities via the VEGAN package (Xu et al., 2023).

## 3 Results

### 3.1 Soil physicochemical properties

**Figure 1:**
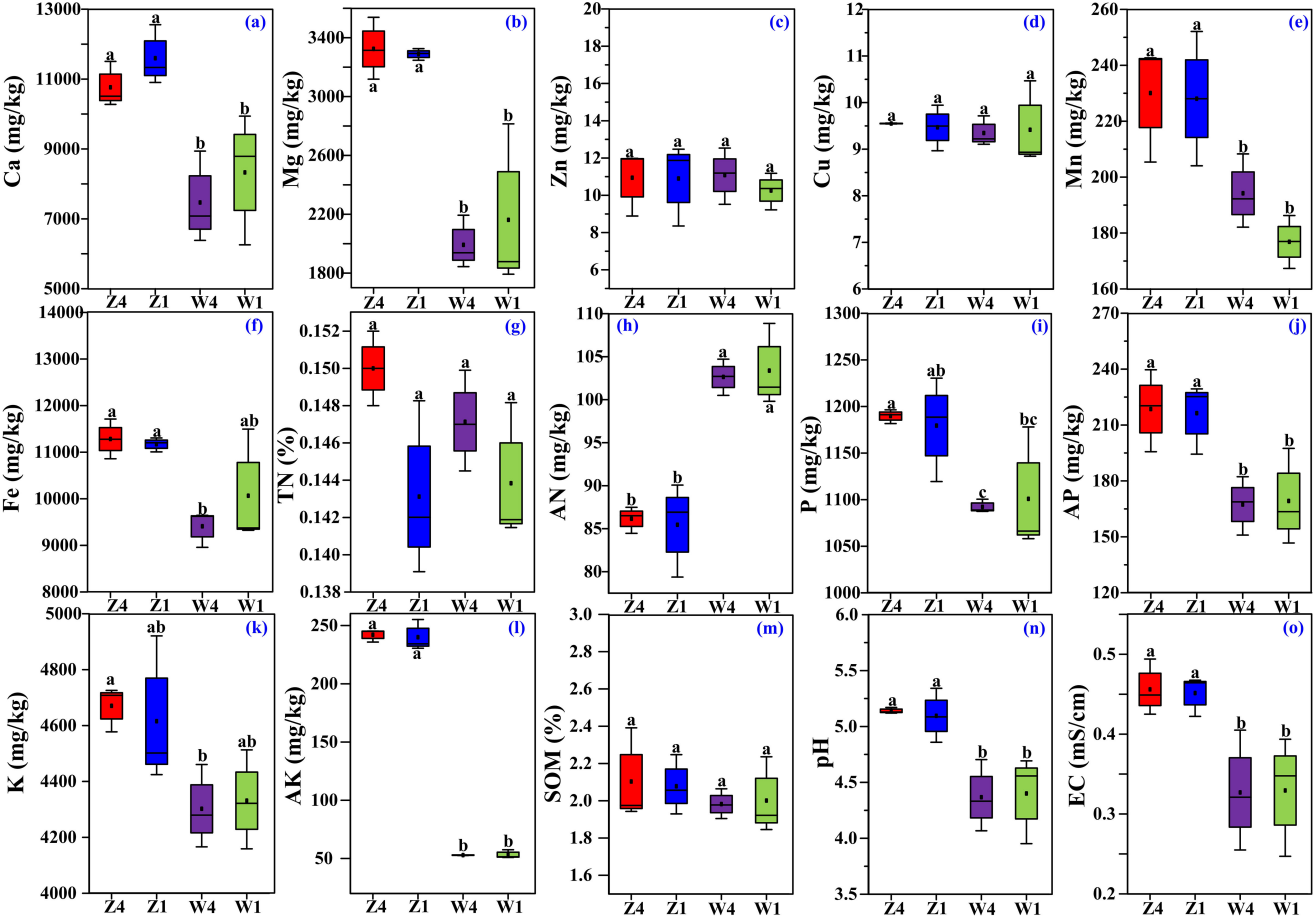
Basic physicochemical properties of rhizosphere soils, including concentrations of Ca **(a)**, Mg **(b)**, Zn **(c)**, Cu **(d)**, Mn **(e)**, Fe **(f)**, total N (TN, **g**), alkaline hydrolyzable N (AN, **h**), total P **(i)**, available P (AP, **j**), total K **(k)**, available K (AK, **l**), and soil organic matter (SOM, **m**), soil pH **(n)** and soil EC **(o)**. Different lowercase letters indicate significant differences (*p* ≤ 0.05, one-way ANOVA with Tukey’s test). Z1, Z4, W1, and W4 represent the rhizosphere soils of variety T1at site Z, variety T4 at site Z, variety T1 at site W, and variety T4 at site W, respectively.

The concentrations of Ca (**Figure 1a**), Mg (**Figure 1b**), Mn (**Figure 1e**), AP (**Figure 1j**), and AK (**Figure 1l**) in the soils from the Z site were significantly higher than those from the W site. Similarly, the Fe concentration in Z1 was significantly higher than in W4 (**Figure 1f**), and the TP (**Figure 1i**) and TK (**Figure 1k**) levels in Z4 were significantly higher than in W4. Similarly, soil pH (**Figure 1n**) and EC (**Figure 1o**) were generally higher at site Z. In contrast, the concentration of soil AN (**Figure 1h**) was significantly lower at the site Z. Most soil physicochemical properties did not significantly differ between the two PSH varieties at the same site (Figure 1c,d,g,m), indicating spatial homogeneity within sites.

### 3.2 Bacterial community structure

A total of 552,960 high-quality sequences were obtained, yielding 42,280 operational taxonomic units (OTUs) with sequencing coverage between 98.10% and 98.78%, indicating that the sequencing depth was sufficient for evaluating microbial diversity. The number of OTUs was significantly higher (*p* < 0.05) in soils from site Z (Z1:3,857; Z4:3,811) than from site W (W1:3,192; W4: 3,233) (**Supplementary Figure S1a**). The Chao1 index also indicated greater microbial diversity in Z soils (**Supplementary Figure S1d**). Principal coordinate analysis (PCoA) revealed distinct differences in the bacterial community structure between the two sites (**Supplementary Figure S1e**). Sequences were classified into 45 phyla and 1,183 genera. The three mostabundant phyla were Proteobacteria (22.56%-29.84%), Chloroflexi (22.89%-24.22%), and Actinobacteria (13.85%-20.50%) (**Supplementary Figure S2a**). The most three abundant genera were *norank_f_norank_o_norank_c_AD3* (AD3, 0.01%-6.80%), *norank_f_norank_o_SBR1031* (*SBR1031*, 1.23%-6.89%), and *norank_f_norank_o_norank_c_KD4-96* (*KD4-96*, 1.86%-2.09%) (**Supplementary Figure S2b**).

**Figure 2:**
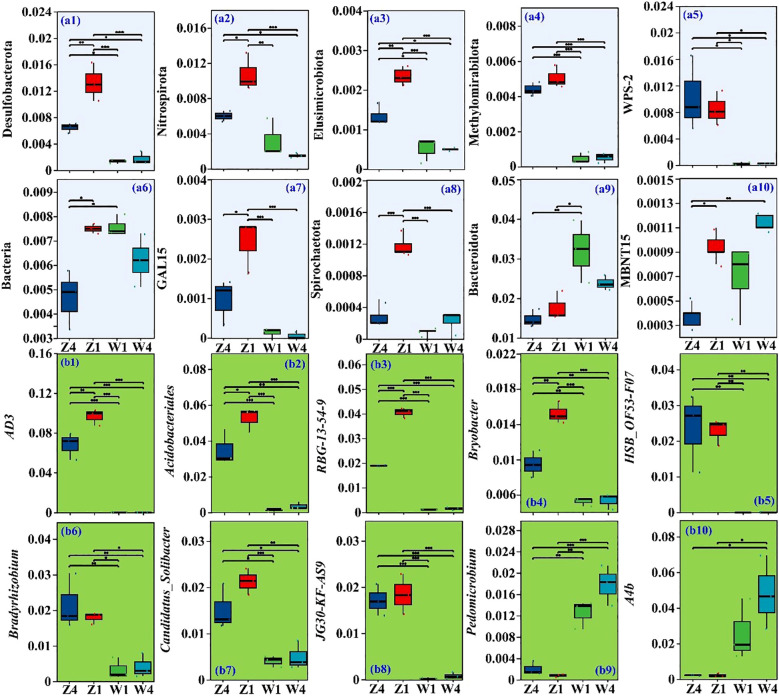
Relative abundances of Top_10_ phyla (a1-a10) and Top_10_ genera (b1-b10). ** and * indicate significant differences at *p* ≤ 0.05 and *p* ≤ 0.01, respectively. Z1, Z4, W1, and W4 are as defined in **Figure 1**.

Thirteen phyla and 385 genera exhibited significant differences in abundance (*p* < 0.05) among different soils from the rhizospheres of various PSH seedlings. The TOP10 differential phyla and genera were selected for further analysis (**Figures 2a1–a10**). The relative abundances of the phyla Desulfobacterota (**Figure 2a1**), Nitrospirota (**Figure 2a2**), Elusimicrobiota (**Figure 2a3**), Methylomirabilota (**Figure 2a4**), WPS-2 (**Figure 2a5**), GAL15 (**Figure 2a7**), and Spirochaetota (**Figure 2a8**) were generally higher in Z soils, particularly for T1. In contrast, the abundances of unclassified_k_norank_d_Bacteria (**Figure 2a6**), Bacteroidota (**Figure 2a9**), and MBNT15 (**Figure 2a10**) were lowest in the T4 rhizosphere soil at site Z.

At the genus level, *AD3* (**Figure 2b1**) was most abundant in Z1, followed by Z4, W1, and W4. Similar patterns were observed for *norank_f_norank_o_Acidobacteriales* (*Acidobacteriales*, **Figure 2b2**), *norank_f_norank_o_RBG-13-54-9* (*RBG-13-54-9*, **Figure 2b3**), and *Bryobacter* (**Figure 2b4**). The relative abundances of *HSB_OF53-F07* (**Figure 2b5**), *Bradyrhizobium* (**Figure 2b6**), *Candidatus Solibacter* (**Figure 2b7**), and *norank f_JG30-KF-AS9* (*AS9*, **Figure 2b8**) were significantly higher in Z soils, especially for T1. Conversely, *Pedomicrobium* (**Figure 2b9**) and *norank_f_A4b* (*A4b*, **Figure 2b10**) were more abundant in the W soils, particularly for T4 variety.

### 3.3 Environmental factors affecting bacterial community structure

**Figure 3:**
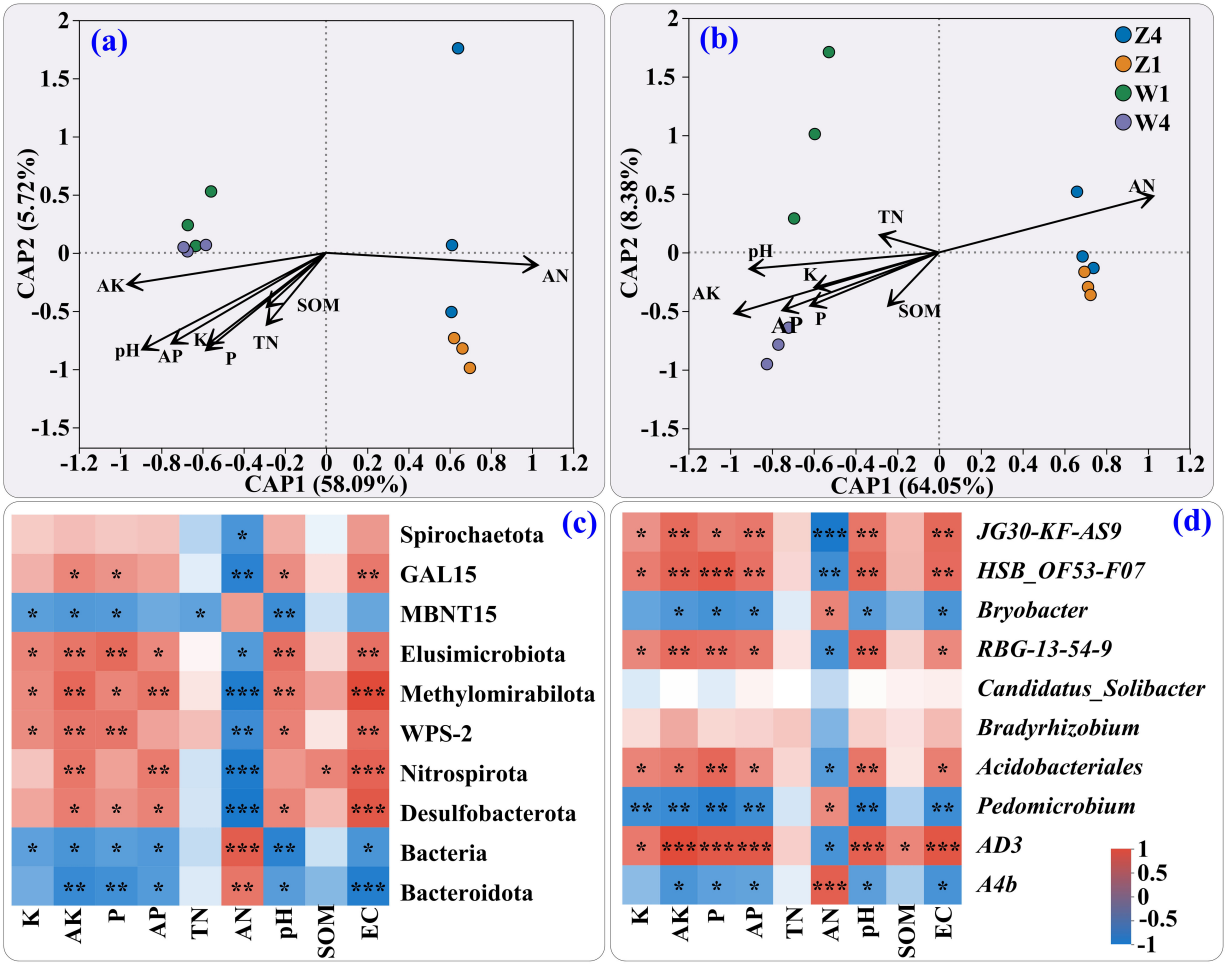
Redundancy analysis (RDA) of the microbial communities and soil factors at the phylum **(a)** and genus **(b)** levels. Heatmaps show correlations between soil properties and dominant bacteria at the phylum **(c)** and genus **(d)** levels. The significance levels indicated as * (0.01 < p ≤ 0.05), ** (0.001 < p ≤ 0.01), and *** (p ≤ 0.001).

Redundancy analysis (RDA) at the phylum level (**Figure 3a**) showed that CAP1 (58.09%) and CAP2 (5.72%) explained 63.81% of the community variation. At the genus level (**Figure 3b**), the ordination axes CAP1 (64.05%) and CAP2 (8.38%) explained 72.43%. AN, AK, and pH were the primary driving factors, with clear separation between Z and W sites.

At the phylum level (**Figure 3c**), the relative abundances of Spirochaetota, GAL15, Elusimicrobiota, Methylomirabilota, WPS-2, Nitrospirota, and Desulfobacterota were negatively correlated with AN but generally positively correlated with AK, pH, and EC except for TN and SOM (which showed no significant correlation). At the genus level (**Figure 3d**), the relative abundances of *AS9, HSB_OF53-F07*, *RBG-13-54-9*, *Acidobacteriales*, and *AD3* were positively correlated with most soil physicochemical properties except TN, AN, and SOM.

### 3.4 Metabolomic analysis of PSH rhizosphere soil samples

**Figure 4:**
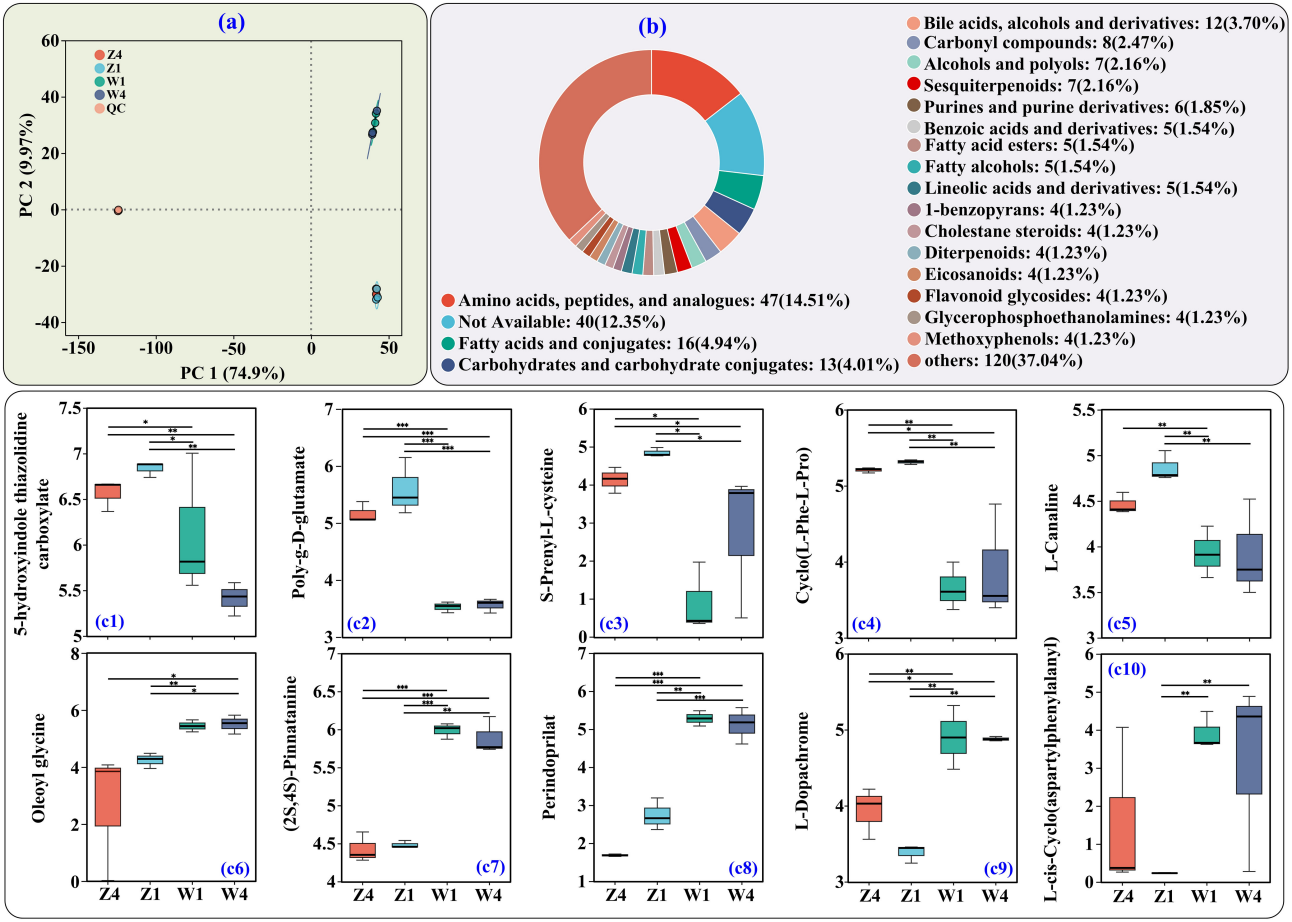
Untargeted metabolomics analysis of PSH rhizosphere soils. **(a)** Principal coordinate analysis (PCoA). **(b)** HMDB classification of differential metabolites (DMs) at the subclass level. (c1–c10) Relative abundances of the top 10 DMs (by VIP value) belonging to amino acids/peptides and analogs. *, **, and *** indicate significance at adjusted *p* < 0.05, < 0.01, and < 0.001, respectively (*n* = 3). Y-axis shows log_10_ transformed relative abundance. Z1, Z4, W1, and W4 are as defined previously.

PCoA of soil metabolites revealed distinct profiles between sites (**Figure 4a**). We identified 383 differential metabolites (DMs, VIP > 1, *p* < 0.05). Based on HMDB classification, the top three categories of DMs were amino acids/peptides and analogs (47 DMs, 14.51%), fatty acids and conjugates (16 DMs, 4.94%), and carbohydrates and carbohydrate conjugates (13 DMs, 4.01%) (**Figure 4b**).

Among the top10 AAs/peptides and analogs (by VIP values), the abundances of 5-hydroxyindole thiazolidine carboxylate (**Figure 4c1**), poly-g-D-glutamate (**Figure 4c2**), S-prenyl-L-cysteine (**Figure 4c3**), cyclo(L-Phe-L-Pro) (**Figure 4c4**), and L-canaline (**Figure 4c5**) were higher at site Z. In contrast, oleoyl glycine (**Figure 4c6**), (2S,4S)-pinnatanine (**Figure 4c7**), perindoprilat (**Figure 4c8**), L-dopachrome (**Figure 4c9**), and L-cis-cyclo(aspartylphenylalanyl) (**Figure 4c10**) were less abundant at site Z.

### 3.5 Metabolomic analysis of PSH tuberous roots

**Figure 5:**
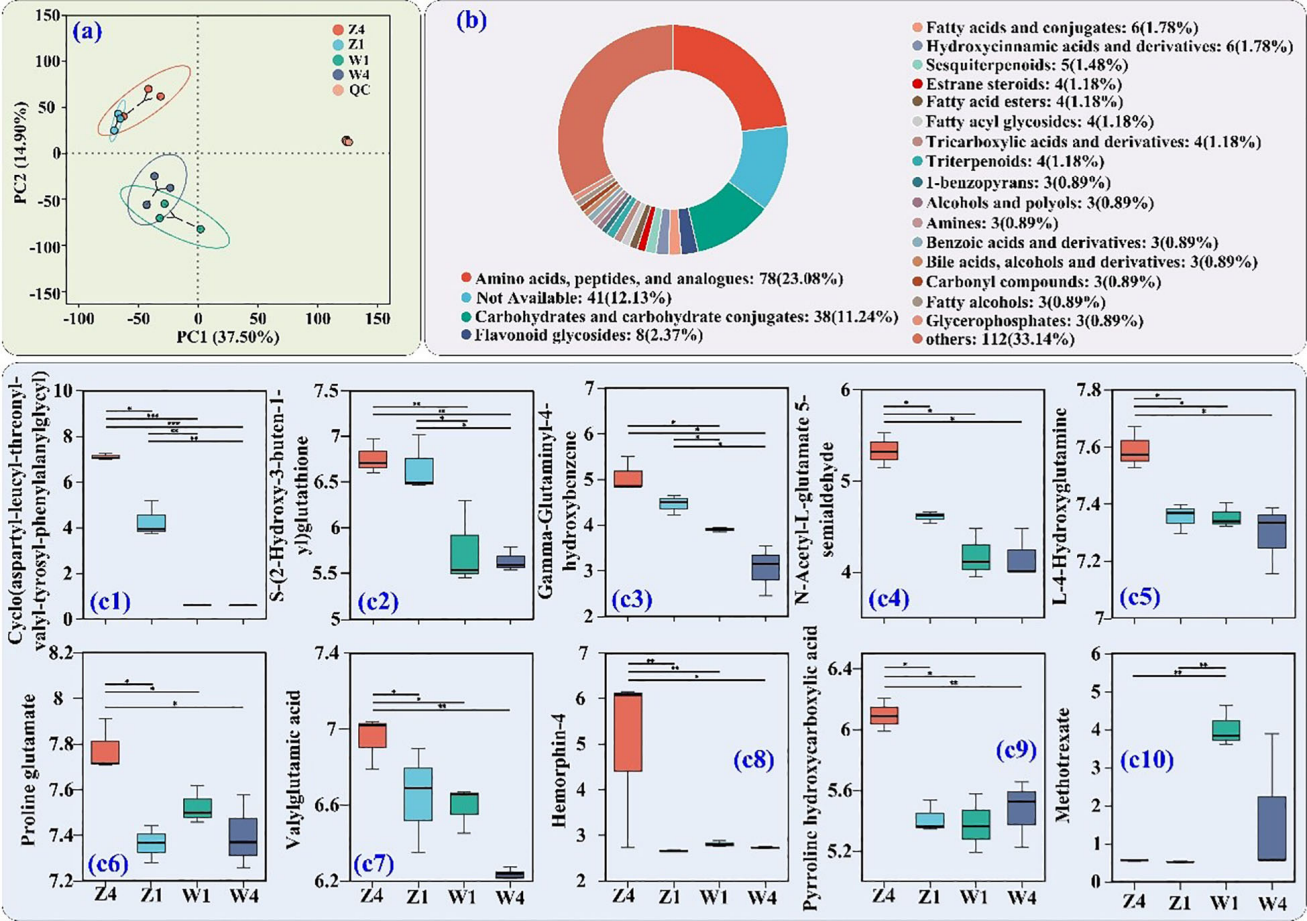
Untargeted metabolomics analysis of PSH tuberous roots. **(a)** PCoA. **(b)** HMDB classification of DMs. (c1–c10) Relative abundance of the top 10 DMs (by VIP value) from amino acids/peptides and analogs class. Significance levels and sample labels as in **Figure 4**.

PCoA of tuberous root metabolites showed clustering by site rather than variety (**Figure 5a**). Among 356 DMs identified, 78 belonged to AAs/peptides and analogs (23.08%), 38 carbohydrates and carbohydrate conjugates (11.24%), and 8 flavonoid glycosides (2.37%) (**Figure 5b**).

The top 10 VIP DMs from the AAs/peptides and analogs class are shown in **Figure 5c1**. The relative abundances of cyclo(aspartyl-leucyl-threonyl-valyl-tyrosyl-phenylalanylglycyl) (**Figure 5c1**), S-(2-hydroxy-3-buten-1-yl) glutathione (**Figure 5c2**), and gamma-glutaminyl-4-hydroxybenzene (**Figure 5c3**) were higher in roots from site Z. Notably, T4 roots from site Z had the highest abundances of these metabolites, as well as N-acetyl-L-glutamate 5-semialdehyde (**Figure 5c4**), L-4-hydroxyglutamine (**Figure 5c5**), proline glutamate (**Figure 5c6**), valylglutamic acid (**Figure 5c7**), hemorphin-4 (**Figure 5c8**), and pyrroline hydroxycarboxylic acid (**Figure 5c9**). Methotrexate (**Figure 5c10**) was more abundant in roots from site W. These results indicate a greater accumulation of key AAs/peptides and analogs in T4 roots from site Z.

### 3.6 Factors in PSH rhizosphere soils affecting the accumulation of key metabolites in PSH tuberous roots

#### 3.6.1 Relationships between bacterial communities and DMs in PSH tuberous roots

**Figure 6:**
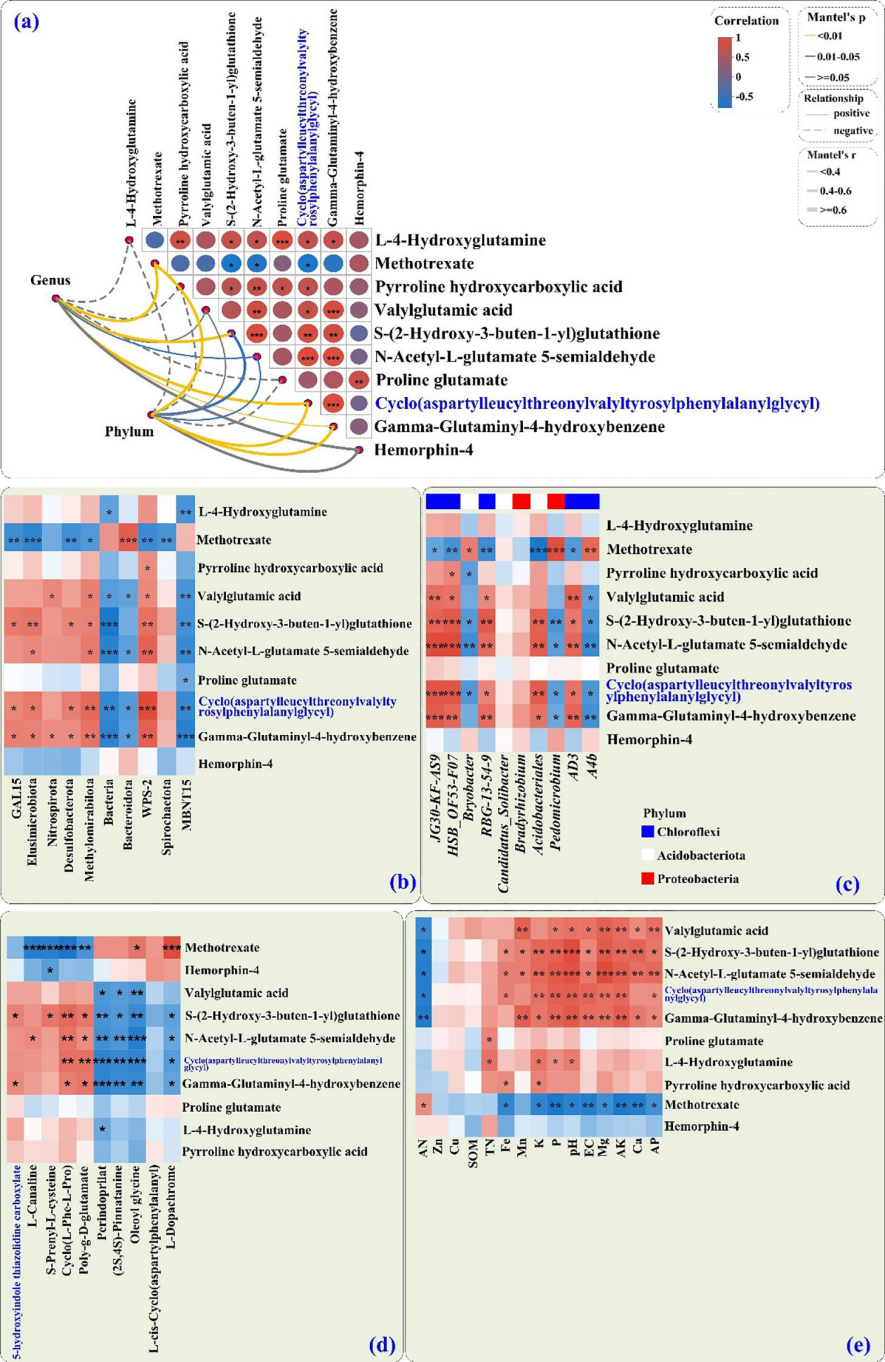
The bacterial community composition (measured by the Bray–Curtis distance) was correlated with metabolites (TOP_10_ VIP values) in PSH tuberous roots on the basis of Mantel tests **(a)**. The edge width represents Mantel’s r statistic results, with larger values of r indicating a stronger response to lead. The type of edge indicates statistical significance, with solid edges representing positive correlations and dashed edges signifying negative correlations. Spearman correlation analysis was conducted to examine the relationships (1) between soil-dominant microbial communities [phylum **(b)** and genus **(c)** levels] and amino acids/peptides and analogs in PSH roots; (2) between the abundance of soil differentially abundant metabolites and amino acids/peptides and analogs in PSH roots **(d)**; and (3) between soil physicochemical properties and amino acids/peptides and analogs in PSH roots **(e)**. The transition from dark blue to light blue indicates a negative correlation, whereas the shift from light red to dark red signifies a positive correlation. Significance levels and sample labels as in **Figure 3**.

Mantel tests revealed significant correlations between soil bacterial communities and the top 10 root AAs/peptides DMs (**Figure 6a**), where the thickness of the lines indicates the strength of the correlations. The absolute value of Mantel’s r statistic was calculated and visualized following the methodology outlined by Quilodrán et al. (2025).

Generally, the abundances of bacterial phyla and genera were positively correlated with methotrexate, valylglutamic acid, S-(2-hydroxy-3-buten-1-yl) glutathione, N-acetyl-L-glutamate-5-semialdehyde, cyclo(aspartyl-leucyl-threonyl-valyl-tyrosyl-phenylalanylglycyl), gamma-glutaminyl-4-hydroxybenzene, and hemorphin-4 (**Figure 6a**), but not significantly correlated (or negatively) with L-4-hydroxyglutamine, pyrroline hydroxycarboxylic acid, and proline glutamate.

Phyla such as GAL15, Elusimicrobiota, Nitrospirota, Desulfobacterota, Methylomirabilota, and WPS-2 were positively correlated with valylglutamic acid, S-(2-hydroxy-3-buten-1-yl) glutathione, N-acetyl-L-glutamate 5-semialdehyde, cyclo(aspartyl-leucyl-threonyl-valyl-tyrosyl-phenylalanylglycyl), and gamma-glutaminyl-4-hydroxybenzene (**Figure 6b**). These DMs were negatively correlated with MBNT15, Bacteroidota, and unclassified_k_norank_d_Bacteria (**Figure 6b**). Methotrexate correlated positively with Bacteroidota but negatively with GAL15, Elusimicrobiota, Desulfobacterota, Methylomirabilota, WPS-2, and Spirochaetota (**Figure 6b**).

*Bryobacter*, *Pedomicrobium*, and *A4b* were positively correlated with the abundance of methotrexate; but methotrexate was negatively correlated with the genera *AS9*, *HSB_OF53-F07*, *RBG-13-54-9*, *Acidobacteriales*, and *AD3* (**Figure 6c**). Meanwhile, S-(2-hydroxy-3-buten-1-yl) glutathione, N-acetyl-L-glutamate 5-semialdehyde, cyclo(aspartyl-leucyl-threonyl-valyl-tyrosyl-phenylalanylglycyl), and gamma-glutaminyl-4-hydroxybenzene were negatively correlated with the genera *Bryobacter*, *Pedomicrobium*, and *A4b* (**Figure 6c**). In contrast, S-(2-hydroxy-3-buten-1-yl) glutathione, N-acetyl-L-glutamate 5-semialdehyde, cyclo(aspartyl-leucyl-threonyl-valyl-tyrosyl-phenylalanylglycyl), and gamma-glutaminyl-4-hydroxybenzene were positively correlated with the genera *AS9*, *HSB_OF53-F07*, *RBG-13-54-9*, *Acidobacteriales*, and *AD3* (**Figure 6c**).

#### 3.6.2 Relationships between key metabolites in soils and those in PSH tuberous roots

The abundances of S-(2-hydroxy-3-buten-1-yl) glutathione, N-acetyl-L-glutamate 5-semialdehyde, cyclo(aspartyl-leucyl-threonyl-valyl-tyrosyl-phenylalanylglycyl), and gamma-glutaminyl-4-hydroxybenzene in the tuberous roots were positively correlated with 5-hydroxyindole thiazolidine carboxylate, L-canaline, S-prenyl-L-cysteine, cyclo(L-Phe-L-Pro), and poly-g-D-glutamate in the rhizosphere soils, but negatively correlated with perindopril, (2S,4S)-pinnatanine, oleoyl glycine, L-cis-cyclo(aspartylphenylalanyl), and L-dopachrome (**Figure 6d**). Methotrexate in tuberous roots was positively correlated with oleoyl glycine and L-dopachrome in soils, but negatively with L-canaline, S-prenyl-L-cysteine, poly-g-D-glutamate, and cyclo -(L-Phe-L-Pro) (**Figure 6d**).

#### 3.6.3 Relationships between soil physicochemical properties and DMs in PSH tuberous roots

Most root DMs were positively correlated with soil pH, EC, TK, AK, TP, AP, Ca, Mg, Mn, and Fe (**Figure 6e**). Exceptions were methotrexate and hemorphin-4, which were generally negatively correlated with these properties, and positively with AN for methotrexate.

## 4 Discussions

### 4.1 Balanced application of N and other fertilizers may optimize the soil bacterial community structure

The number of OTUs in our PSH rhizosphere soils (3,192–3,811, **Supplementary Figure S1a**) exceeded those reported by Liu et al. (2024) for Zherong soils (1,971–2,406). Both OTUs number (**Supplementary Figure S1a**) and the Chao index (**Supplementary Figure S1d**) were significantly higher at site Z than site W, consistent with reports of site-dependent bacterial diversity in other plants like *Lycium barbarum* L (Liu et al., 2022).

In this study, the more diverse soil bacterial community structure at the Z site compared to the W site (**Supplementary Figures S1a, d**) may be attributed to 1) A moderate level of soil nitrogen. The concentration of AN was significantly lower in site Z than in site W (**Figure 1h**), which was negatively correlated with most phyla (**Figure 3c**) and genera (**Figure 3d**). The soil AN concentration, ranging from 50 to 100 mg·kg^-1^, is moderate and may effectively support crop growth (Ma et al., 2024). However, high soil AN concentrations can inhibit N absorption by plants, thereby reducing AAs accumulation (Tian et al., 2020). 2) Higher concentrations of Ca (**Figure 1a**), Mg (**Figure 1b**), Mn (**Figure 1e**), AP (**Figure 1j**), and AK (**Figure 1l**), as well as higher pH (**Figure 1n**) and EC (**Figure 1o**) at site Z. Low soil pH can lead to ion leaching (Neina, 2019). Therefore, the generally lower concentrations of the above ions in the W soil than in the Z soil may have resulted from the lower soil pH in the W soil (**Figure 1n**). In addition, these parameters were generally positively correlated with the abundance of most top 10 phyla (**Figure 3c**) and TOP10 genera (**Figure 3d**). Reports have also suggested that relatively high levels of these essential elements, such as Ca, Mg, Mn, P, and K, benefit the development of soil microbial communities and plant growth (Dincă et al., 2022). The above results explained why the values of the OTUs and the Chao1 index in the soils at the Z site were significantly greater than those at the W site.

### 4.2 Predominant bacteria at the Z site may create favorable conditions for PSH growth

Desulfobacterota contribute to the soil sulfur cycle by utilizing organic matter as an electron donor for sulfate reduction, aiding decomposition and mineralization (Dyksma and Pester, 2024). Strains belonging to the Nitrospirota phylum participate in the soil N cycle by oxidizing nitrite (NO_2_^–^) to form nitrate (NO_3_^–^), which may increase N availability in the soil and subsequently increase N uptake by plants (Koch et al., 2014). Elusimicrobiota participate in the biosynthesis of glycans, AAs, B vitamins, and nicotinamide (Pi et al., 2022). WPS-2 phylum members (a group of anoxygenic photosynthetic bacteria) participate in photosynthesis, thereby facilitating soil carbon cycling (Ward et al., 2019). The enrichment of strains belonging to the above phyla may increase the nutrient cycle in soil, ultimately promoting soil fertility (Harindintwali et al., 2021). The high abundance of the phyla Desulfobacterota (**Figure 2a2**), Nitrospirota (**Figure 2a2**), and Elusimicrobiota (**Figure 2a3**) at the Z site was positively correlated with the growth of PSH.

Acidobacteria are known for degrading complex carbohydrates, nutrient cycling, extracellular polysaccharide (EPSs) synthesis in soil (Costa et al., 2020), and producing plant growth-promoting substances like indole-3-acetic acid and siderophores, thereby promoting plant growth (Kuramae and De Assis Costa, 2019). In this study, the *Acidobacteriales* genus was one of the dominant bacterial genera in PSH rhizosphere soils, and the genera *Acidobacteriales* (**Figure 2b2**), *Bradyrhizobium* (**Figure 2b6**), *Candidatus Solibacter* (**Figure 2b7**), and *AS9* (**Figure 2b8**) all belong to the Acidobacteria phylum. The greater abundance of these bacteria in Z soil may have created more favorable growth conditions for PSH than those from the W site. The importance of Acidobacteria in balancing the PSH rhizosphere microbiome has been emphasized previously (Wu et al., 2019; Liu et al., 2024).

### 4.3 High abundance of AAs/peptides and *analogs* in soils *does* not always result in high accumulation in *the* tuberous roots of PSH

Microbial communities significantly influenced soil DM composition, which was highly similar within sites (**Figure 4a**). AAs/peptides and analogs were the most abundant soil DMs (47 DMs, **Figure 4b**), many with plant growth-promoting functions. For instance, poly-g-D-glutamate (**Figure 4c2**), primarily of bacterial origin, can enhance crop nutrient uptake and soil water retention and fertility in soil (Zhang et al., 2017). Cyclo(L-Phe-L-Pro) (**Figure 4c4**) is a cyclic dipeptide that microorganisms can secrete to alleviate aluminum stress in wheat by promoting root growth and alleviating oxidative damage (Wang et al., 2024). L-canaline (**Figure 4c5**) may increase plant resistance to insect and pathogen attacks (Rosenthal, 2001). The higher abundance of these beneficial metabolites in Z soils likely supported better PSH growth at that site.

While AAs and peptides are key PSH active constituents (Sha et al., 2023), a high abundance of these DMs in soil did not directly translate to high accumulation in roots. For example, Z4 soil had 31 AAs/peptides DMs with higher abundance *vs*. 16 in Z1 soil, but Z4 roots had 45 such DMs *vs*. 33 in Z1 roots (**Supplementary Figures S4**, **S5**). Interestingly, Z soils generally had a lower abundance of these DMs than W soils (**Supplementary Figure S4**), yet T4 roots from site Z accumulated the highest levels (**Supplementary Figure S5**). This suggests that soil DM abundance alone is not predictive of root accumulation, and other factors (e.g., plant uptake, translocation, and metabolism) play critical roles.

### 4.4 DMs with high VIP values tended to accumulate in the tuberous roots of T4 from the Z site

In this study, the tuberous roots of T4 from site Z accumulated a significantly higher level of high-VIP DMs with medicinal relevance. For example, cyclo(aspartyl-leucyl-threonyl-valyl-tyrosyl-phenylalanylglycyl) (**Figure 5c1**), a cyclic peptide that can (1) inhibit lipopolysaccharide-induced inflammation and apoptosis by modulating the phosphatidylinositol 3-kinase/protein kinase B (*PI3K/Akt*) signaling pathway and (2) exhibit anticancer activity against esophageal, pancreatic, lung, and colon cancers (You et al., 2021; Tantai et al., 2016). Additionally, gamma-glutaminyl-4-hydroxybenzene (**Figure 5c3**) and N-acetyl-L-glutamate-5-semialdehyde (**Figure 5c4**) in tuberous roots displayed similar trends. Gamma-glutaminyl-4-hydroxybenzene, a phenolic amino acid, is recognized as a significant phenolic compound found in mushrooms (Sinha et al., 2025). N-Acetyl-L-glutamate-5-semialdehyde, an intermediate in arginine biosynthesis, is linked to L-proline metabolism during oxidative stress (Tian et al., 2024). L-proline is known to increase plant stress tolerance by regulating amino acid metabolism under drought and salinity conditions (Alvarez et al., 2022). The optimal soil conditions at site Z, particularly for T4 cultivation, therefore appear to promote the accumulation of these high-value medicinal compounds.

### 4.5 Correlation analysis of soil physicochemical properties, soil AA-related DMs, soil bacterial populations, and AA-related DMs in PSH tuberous roots

Soil microecological conditions significantly influence plant secondary metabolism (Chiappero et al., 2021). Our correlation analysis (**Figure 6**) integrated soil properties, bacterial communities, and root metabolites. Plant growth-promoting rhizobacteria (PGPR) may increase plant growth and crop yield and quality (Chiappero et al., 2021). For example, rhizosphere-associated bacteria may promote the biosynthesis of secondary metabolites in *Astragalus membranaceus* (Li et al., 2021) and *basil* (Khalediyan et al., 2021). In this study, beneficial root DMs such as cyclo(aspartyl-leucyl-threonyl-valyl-tyrosyl-phenylalanylglycyl), N-acetyl-L-glutamate 5-semialdehyde, and gamma-glutaminyl-4-hydroxybenzene correlated positively with TK, AK, TP, AP, Mg, Mn, and Fe but negative with AN in rhizosphere soils (**Figure 6e**). The appropriate K supply enhanced photoassimilate transport from leaves to roots and increased nitrogen use efficiency by influencing photosynthesis, C and N metabolizing enzyme activities, nitrate assimilation gene activities, and nitrate transport (Xu et al., 2020). Phosphorus limitation induces significant alterations in the carbohydrate metabolic pathways of plants, resulting in sugar accumulation and the activation of stress response mechanisms and the inhibition of amino acids synthesis, while adequate phosphorus supply can reverse this situation (Wei et al., 2025). Therefore, maintaining a relatively abundant supply of P and K in the soil is conducive to the accumulation of amino acids and peptides in PSH.

Most of the DMs showed positive correlations with specific bacterial phyla (**Figure 6b**) and genera (**Figure 6c**), including *AS9*, *Acidobacteriales*, *HSB_OF53-F07*, and *AD3* (**Figure 6c**). It is well known that soil microorganisms can enhance the metabolism of plant secondary metabolites by regulating soil enzyme activity, synthesizing phytohormones, and activating plant signaling pathways—processes that may ultimately promote the synthesis of medicinal compounds (Korenblum et al., 2022). For example, many strains within the phylum Acidobacteriota harbor numerous genes linked to plant growth-promoting traits (PGPTs), which are involved in N fixation, phosphorus solubilization, extracellular polysaccharide production, and siderophore synthesis (Gonçalves et al., 2024). Moreover, soil microorganisms can synthesize precursors of secondary metabolites that serve as essential building blocks for the accumulation of active substances in plants (Korenblum et al., 2022). In this study, several microbially derived compounds, such as poly-g-D-glutamate and cyclo(L-Phe-L-Pro), were positively correlated with multiple DMs in PSH tuberous roots (**Figure 6d**). In addition, the plant proline transporter (ProT) is upregulated under salt stress, thereby promoting the accumulation of proline and other amino acids (Mishra et al., 2024). This may partially explain the positive correlation observed in this study between rhizosphere soil electrical conductivity (EC) and the amino acid accumulation in the tuberous roots.

These findings suggest that soils with moderate nitrogen levels but relatively high concentrations of potassium, phosphorus, and iron may facilitate (1) the enrichment of specific bacterial taxa and (2) the accumulation of key DMs—particularly amino acids, peptides, and their analogs—in PSH tuberous roots.

## 5 Conclusion

The rhizosphere soil of PSH from the Z site exhibited significantly higher concentrations of Ca, Mg, Mn, AP, and AK, as well as higher pH and EC, compared to the W site. Although soil AN levels were significantly lower at the Z site, the bacterial diversity in the PSH rhizosphere soil was significantly greater than that at the W site, with higher relative abundances of Desulfobacterota, Nitrospirota, Elusimicrobiota, and WPS-2. Amino acids, peptides, and analogs constituted the most abundant class of DMs in both the rhizosphere soil and the tuberous roots of PSH. Among these, compounds such as cyclo(aspartyl-leucyl-threonyl-valyl-tyrosyl-phenylalanylglycyl), N-acetyl-L-glutamate 5-semialdehyde, and S-(2-hydroxy-3-buten-1-yl) glutathione were more abundant at the Z site, particularly in the roots of the T4 variety. The PSH rhizosphere soil from the Z site also supported greater bacterial diversity and higher abundance of genera such as *AS9*, *Acidobacteriales*, and *AD3*. These microbial and edaphic characteristics may contribute to the accumulation of AAs-related DMs in the tuberous roots. Collectively, these results provide a theoretical basis for selecting high-quality cultivation areas for PSH and for future quality improvement strategies through targeted regulation of soil microbial communities.

## Data Availability

The data presented in this study are deposited in the NCBI repository under the accession number PRJNA1370052.
