# Supplementary material for: Soil factors enhance amino acid and peptide accumulation in tuberous roots of Pseudostellaria heterophylla
Source: Front Plant Sci. 2025 Dec 3;16:1728201. doi: 10.3389/fpls.2025.1728201 (PMC12710503; doi:10.3389/fpls.2025.1728201)
Supplement: Supplementary file 1 [file SupplementaryFile1.docx]

**Fig. S1** OUT numbers of bacteria in all rhizosphere soils (a). Alpha and Beta diversity indexes of 16S rRNA bacterial libraries, including Chao index (b), Simpson index (c), Shannon index (d), and principal coordinates analysis (PCoA) of soil samples collected from different sites (e). ** and * above the bars indicate significant differences between different sites at the *p* ≤ 0.01 and *p* ≤ 0.05 level, respectively. The abbreviations of T4_Z, T1_Z, T1_W and T4_W indicate the rhizosphere soil samples of T4 variety (Z site), T1 variety (Z site), T1 variety (W site), and T4 variety (W site), respectively.

**Fig. S2** The relative abundance of predominant bacteria at the phylum level (a) and genus level (b) in soil samples collected from different sites. The abbreviations of T4_Z, T1_Z, T1_W and T4_W indicate the rhizosphere soil samples of T4 variety (Z site), T1 variety (Z site), T1 variety (W site), and T4 variety (W site), respectively.

**Fig.** **S3** Volcano maps of differential metabolites (DMs). The abbreviations of T4_Z, T1_Z, T1_W and T4_W indicate the rhizosphere soil samples of T4 variety (Z site), T1 variety (Z site), T1 variety (W site), and T4 variety (W site), respectively.

**Fig. S4** Heatmap tree diagram for 47 differential metabolites (DMs) belonged to amino acids, peptides, and conjugates in the rhizosphere soil of *P. heterophyllae*. Z1, Z4, W1 and W4 indicate the rhizosphere soil of T1 variety at the Z site, T4 variety at the Z site, T1 variety at the W site, and T4 variety at the W site, respectively.

**Fig. S5** Heatmap tree diagram for 78 differential metabolites (DMs) belonged to amino acids, peptides, and conjugates in the tuberous roots of *P. heterophyllae*. Z1, Z4, W1 and W4 indicate the tuberous roots of T1 variety at the Z site, T4 variety at the Z site, T1 variety at the W site, and T4 variety at the W site, respectively.

Fig. S6 QC Sample Evaluation Chart

Table. S1 Quality Control Parameters for Untargeted Metabolomics (Top 100). Metabolite: Names of the metabolites identified in this project. Among them, "metab_[0,9]" denotes metabolites that have not been qualitatively confirmed to specific ones. M/Z: Mass-to-charge ratio, referring to the ratio of the mass of a charged ion to the charge it carries. Retention time: The retention time of a compound in chromatography. Mass error: Mass error (ppm). RSD: Relative standard deviation of QC samples.

**Fig. S1**


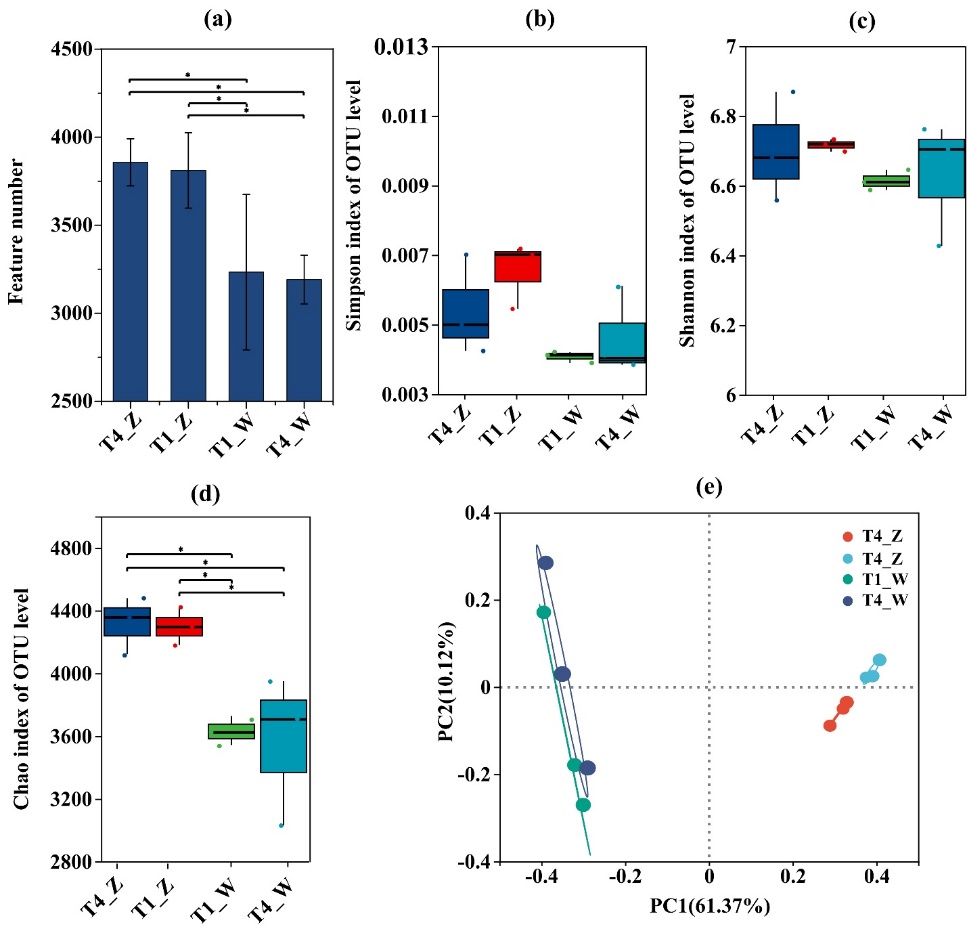


**Fig. S2**


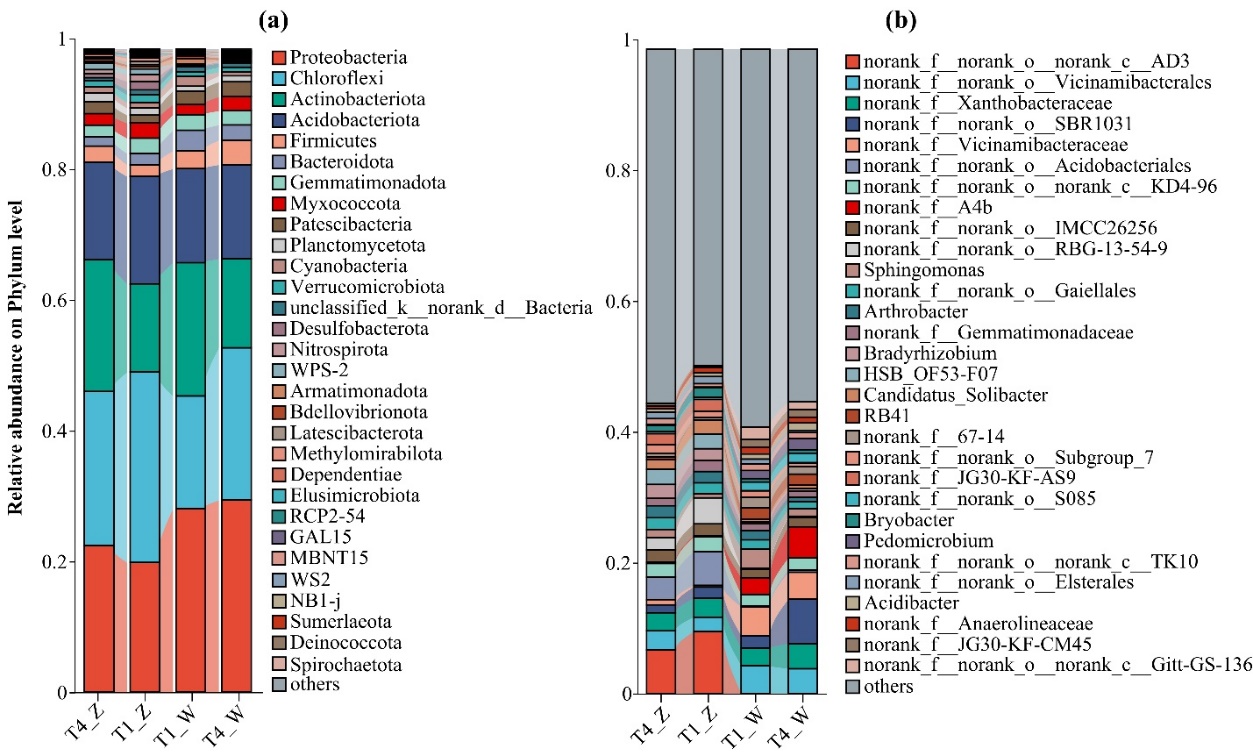


**Fig. S3**


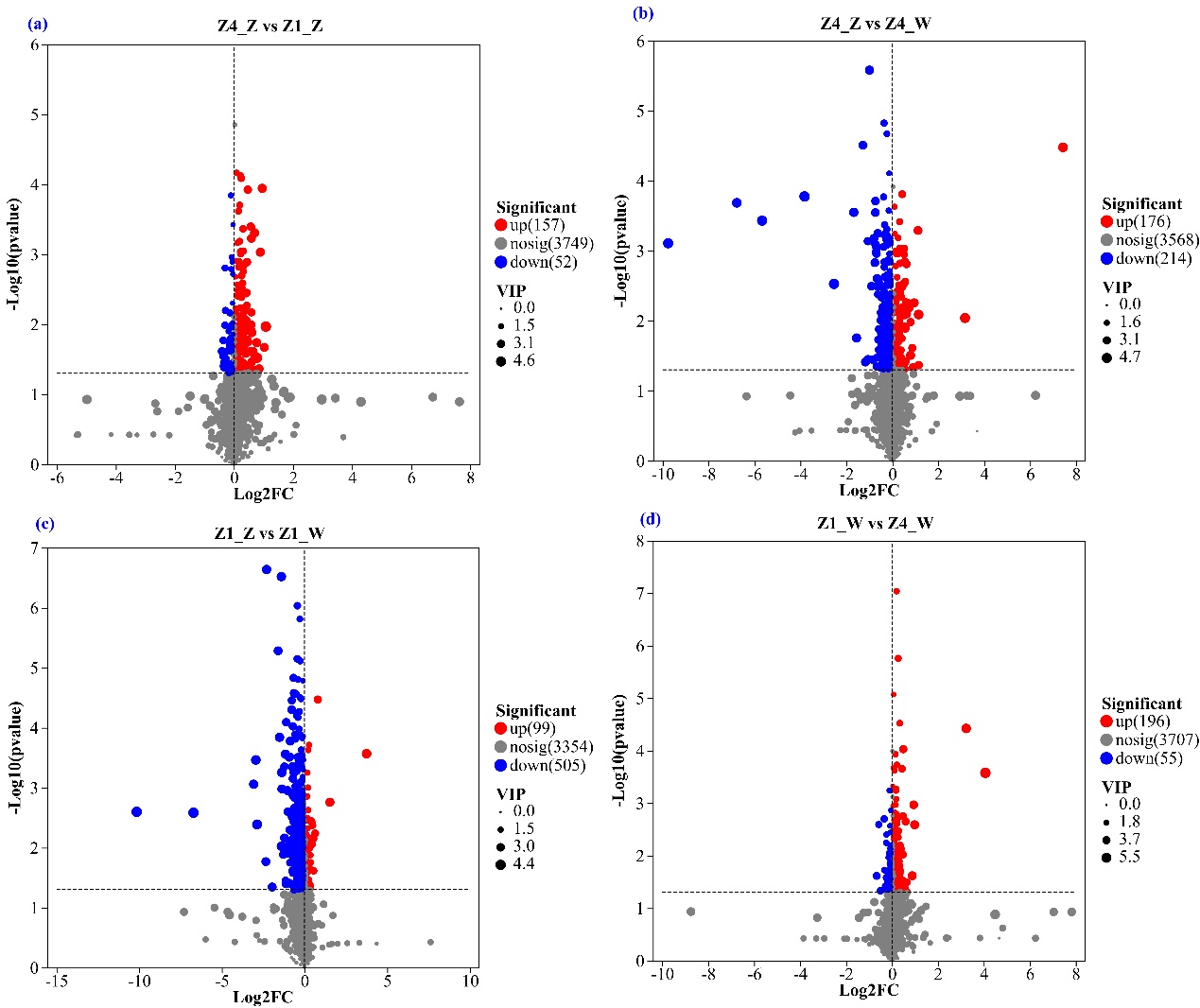


**Fig. S4**


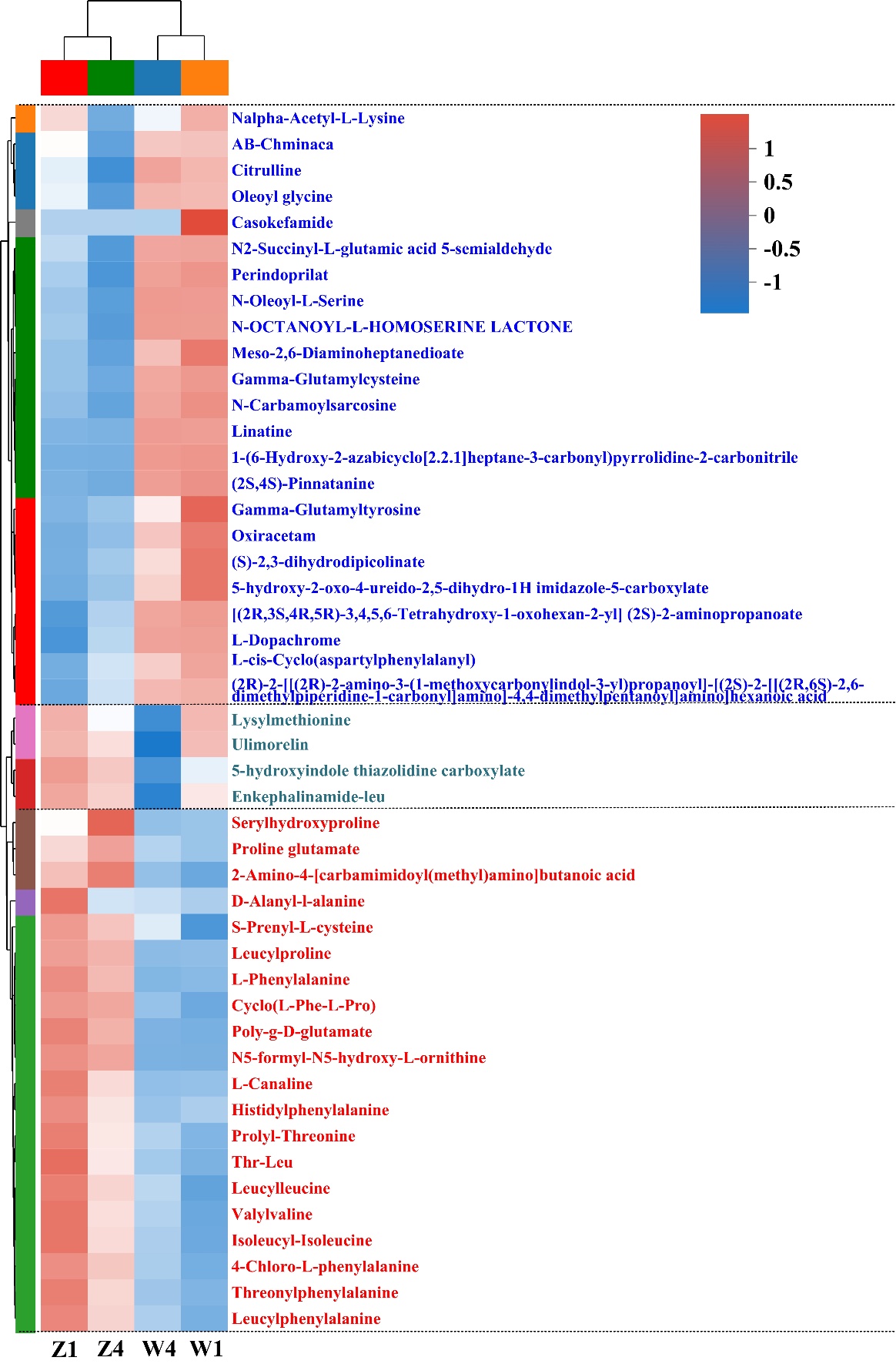


**Fig. S5**


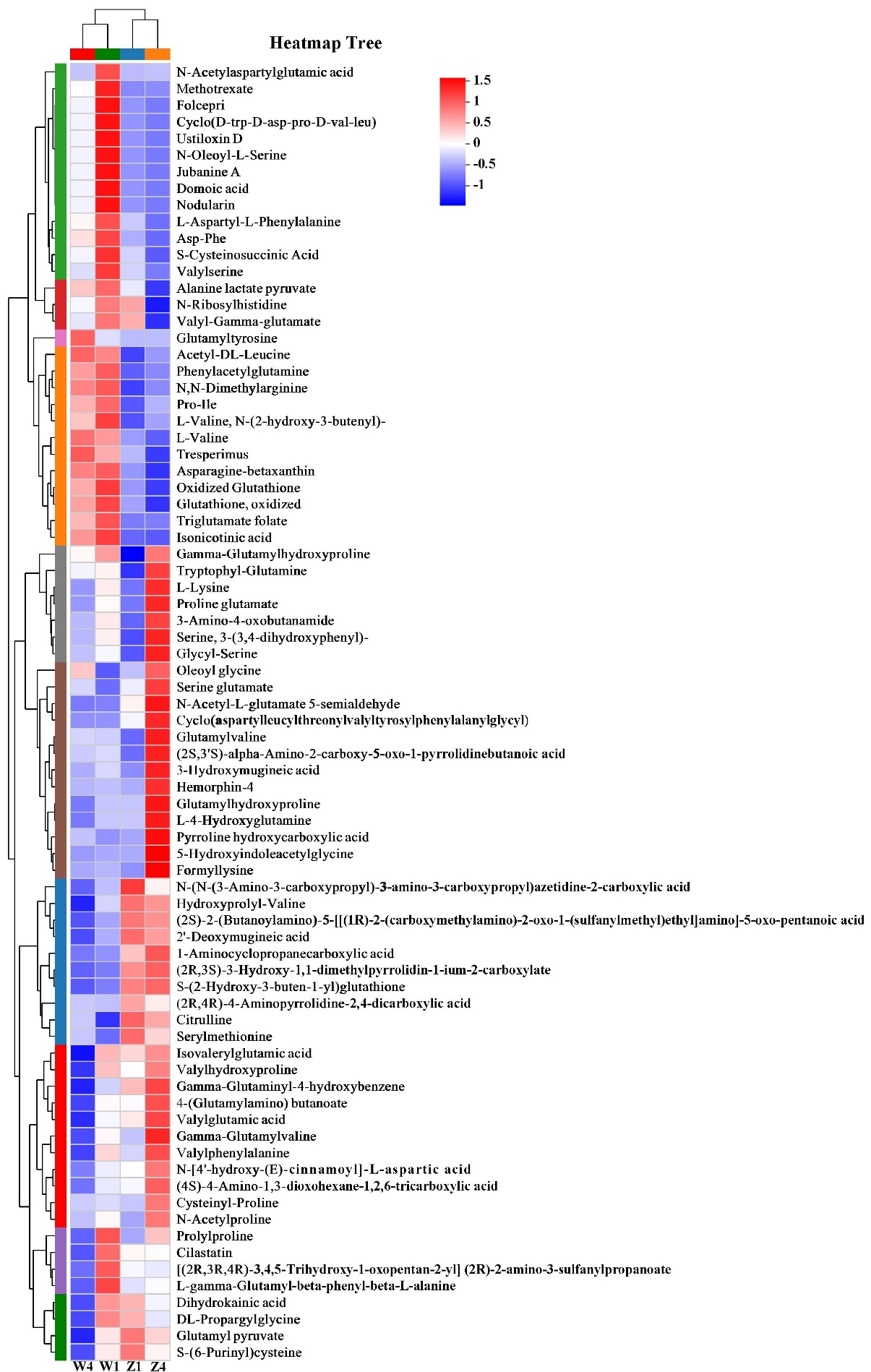


**Fig. S6**


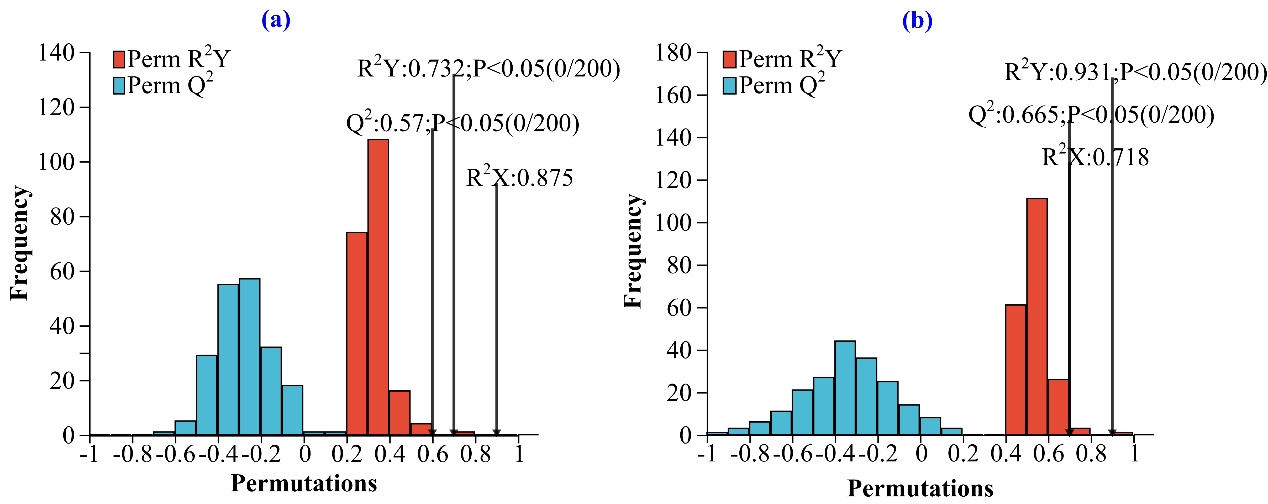


**Table S1**

| **Metabolite** | **M/Z** | **Retention time** | **Mass Error** | **RSD** | **Z4_1** | **Z4_2** | **Z4_3** | **Z1_1** | **Z1_2** | **Z1_3** | **W1_1** | **W1_2** | **W1_3** | **W4_1** | **W4_2** | **W4_3** | **QC01** | **QC02** | **QC03** | **QC04** |
| --- | --- | --- | --- | --- | --- | --- | --- | --- | --- | --- | --- | --- | --- | --- | --- | --- | --- | --- | --- | --- |
| Cyclohexane | 102.128 | 6.3819 | 2.977503021 | 0.004081 | 8.66 | 8.695 | 8.643 | 8.7 | 8.676 | 8.678 | 8.716 | 8.702 | 8.703 | 8.672 | 8.711 | 8.673 | 8.615 | 8.613 | 8.617 | 8.615 |
| Isobutylidene | 175.1187 | 0.5825 | -1.382015847 | 0.00325 | 8.628 | 8.819 | 8.882 | 8.826 | 8.792 | 8.909 | 8.576 | 8.425 | 8.585 | 8.72 | 8.62 | 8.625 | 8.452 | 8.454 | 8.453 | 8.455 |
| Dioctyl succinate | 427.3777 | 6.4969 | -1.100740948 | 0.009124 | 8.525 | 8.483 | 8.507 | 8.548 | 8.54 | 8.533 | 8.584 | 8.494 | 8.556 | 8.534 | 8.606 | 8.561 | 8.417 | 8.413 | 8.408 | 8.416 |
| (+/-)-(Z)-2-(5-Tetradecenyl)cyclobutanone | 282.2787 | 6.2669 | -1.747173778 | 0.016167 | 7.938 | 7.946 | 7.912 | 7.988 | 7.987 | 7.907 | 7.989 | 7.98 | 7.958 | 7.969 | 8.061 | 7.943 | 7.909 | 7.895 | 7.894 | 7.895 |
| Indoleacrylic acid | 188.0704 | 2.5545 | -1.273321536 | 0.002868 | 7.872 | 7.48 | 7.969 | 7.604 | 7.761 | 7.782 | 7.612 | 7.608 | 7.924 | 8.16 | 7.457 | 7.968 | 7.538 | 7.536 | 7.536 | 7.535 |
| Gamma-Aminobutyric acid-betaxanthin | 338.1342 | 3.2035 | -1.488986584 | 0.006542 | 7.74 | 7.072 | 8.075 | 7.558 | 7.877 | 7.789 | 7.733 | 7.815 | 7.77 | 6.396 | 7.608 | 6.625 | 7.49 | 7.487 | 7.486 | 7.483 |
| Chlorobenzene | 154.0416 | 2.6245 | -1.877165208 | 0.005785 | 7.681 | 7.755 | 7.673 | 7.742 | 7.729 | 7.721 | 7.769 | 7.762 | 7.746 | 7.712 | 7.771 | 7.735 | 7.654 | 7.655 | 7.657 | 7.66 |
| Sclareol oxide | 280.2631 | 6.1977 | -1.467784718 | 0.007811 | 7.676 | 7.679 | 7.648 | 7.706 | 7.756 | 7.621 | 7.701 | 7.721 | 7.673 | 7.712 | 7.782 | 7.632 | 7.621 | 7.613 | 7.614 | 7.614 |
| Lauryldiethanolamine | 274.2736 | 5.7494 | -1.574554129 | 0.007047 | 7.627 | 7.662 | 7.57 | 7.624 | 7.605 | 7.598 | 7.645 | 7.624 | 7.678 | 7.588 | 7.645 | 7.6 | 7.452 | 7.45 | 7.448 | 7.445 |
| Butylisopropylamine | 116.1435 | 7.08 | 0.942950129 | 0.003802 | 7.617 | 7.669 | 7.606 | 7.659 | 7.632 | 7.633 | 7.677 | 7.674 | 7.663 | 7.636 | 7.674 | 7.635 | 7.588 | 7.586 | 7.586 | 7.589 |
| L-4-Hydroxyglutamine | 163.0711 | 0.7042 | -1.137763787 | 0.004188 | 7.573 | 7.671 | 7.527 | 7.397 | 7.296 | 7.368 | 7.339 | 7.403 | 7.32 | 7.155 | 7.387 | 7.334 | 7.508 | 7.506 | 7.509 | 7.51 |
| L-Glutamic Acid | 130.0498 | 0.7042 | -0.417971445 | 0.008227 | 7.536 | 7.564 | 7.423 | 7.329 | 7.385 | 7.449 | 7.426 | 7.48 | 7.373 | 7.337 | 7.416 | 7.345 | 7.246 | 7.254 | 7.253 | 7.252 |
| L-Glutamine | 147.0763 | 0.6049 | -0.880155747 | 0.007962 | 7.519 | 7.482 | 7.214 | 6.941 | 7.241 | 7.014 | 6.987 | 6.954 | 7.068 | 7.236 | 6.975 | 6.859 | 7.128 | 7.126 | 7.129 | 7.134 |
| Prolyl-Glutamate | 245.1129 | 0.976 | -1.113355497 | 0.00592 | 7.518 | 7.59 | 7.474 | 7.417 | 7.356 | 7.531 | 7.516 | 7.593 | 7.401 | 7.358 | 7.499 | 7.437 | 7.278 | 7.278 | 7.274 | 7.273 |
| 3-Deoxyglucosone | 127.0389 | 0.6732 | -0.162302404 | 0.003515 | 7.515 | 7.489 | 7.437 | 7.645 | 7.563 | 7.595 | 7.63 | 7.612 | 7.533 | 7.594 | 7.593 | 7.635 | 7.372 | 7.374 | 7.371 | 7.371 |
| 1-Kestose | 522.2025 | 0.7274 | -0.789169799 | 0.001608 | 7.475 | 7.328 | 7.254 | 7.431 | 7.539 | 7.429 | 7.381 | 7.263 | 7.487 | 7.517 | 7.386 | 7.476 | 7.18 | 7.18 | 7.18 | 7.179 |
| L-Valine | 118.0863 | 0.8516 | 0.808147185 | 0.003455 | 7.45 | 7.426 | 7.436 | 7.461 | 7.5 | 7.429 | 7.532 | 7.547 | 7.581 | 7.678 | 7.509 | 7.528 | 7.414 | 7.415 | 7.416 | 7.417 |
| L-Glutamate | 148.0602 | 0.6198 | -1.603654256 | 0.007954 | 7.439 | 7.387 | 7.304 | 7.147 | 7.47 | 7.162 | 7.203 | 7.265 | 7.304 | 7.205 | 7.232 | 7.143 | 7.164 | 7.168 | 7.164 | 7.172 |
| Resiniferatoxin | 667.2281 | 0.6811 | -3.58640874 | 0.00788 | 7.422 | 7.431 | 7.377 | 7.586 | 7.484 | 7.556 | 7.435 | 7.386 | 7.17 | 7.308 | 7.437 | 7.417 | 7.209 | 7.205 | 7.201 | 7.204 |
| Norleucine | 132.1018 | 1.7552 | -0.746454811 | 0.004655 | 7.413 | 6.95 | 7.679 | 7.308 | 7.523 | 7.27 | 7.688 | 7.611 | 7.843 | 8.161 | 7.499 | 7.924 | 7.552 | 7.552 | 7.555 | 7.556 |
| Cyclopropyl-[(3S,4S)-3-(dimethylamino)-4-(hydroxymethyl)piperidin-1-yl]methanone | 227.1751 | 2.5933 | -1.336615904 | 0.001251 | 7.385 | 7.453 | 7.37 | 7.453 | 7.434 | 7.428 | 7.462 | 7.46 | 7.437 | 7.406 | 7.465 | 7.416 | 7.378 | 7.377 | 7.377 | 7.378 |
| Glutamylhydroxyproline | 261.1079 | 0.7042 | -1.000014182 | 0.007663 | 7.344 | 7.449 | 7.322 | 7.16 | 7.058 | 7.112 | 7.087 | 7.175 | 7.07 | 6.912 | 7.148 | 7.079 | 7.308 | 7.307 | 7.305 | 7.313 |
| Pyroglutamic Acid | 130.0498 | 0.612 | -0.453895235 | 0.009448 | 7.334 | 7.301 | 7.057 | 6.821 | 7.134 | 6.873 | 6.89 | 6.883 | 6.966 | 7.057 | 6.875 | 6.769 | 6.968 | 6.965 | 6.968 | 6.975 |
| Sesamose | 684.2552 | 0.8128 | -0.692821647 | 0.006269 | 7.328 | 7.309 | 7.222 | 7.452 | 7.37 | 7.419 | 7.354 | 7.29 | 7.189 | 7.271 | 7.373 | 7.337 | 7.054 | 7.056 | 7.054 | 7.05 |
| Cyclosquamosin C | 900.4946 | 5.8573 | 9.982521278 | 0.007421 | 7.256 | 7.204 | 6.47 | 6.22 | 6.015 | 6.284 | 5.815 | 5.805 | 4.255 | 6.04 | 5.947 | 6.38 | 7.032 | 7.039 | 7.038 | 7.035 |
| 4-Oxo-L-proline | 147.0762 | 0.7042 | -1.680722098 | 0.007096 | 7.238 | 7.249 | 7.063 | 6.934 | 7.063 | 7.068 | 7.022 | 7.019 | 7.007 | 6.994 | 7.012 | 6.943 | 6.925 | 6.923 | 6.926 | 6.931 |
| Withanolide B | 419.2608 | 7.2494 | 6.003067378 | 0.002375 | 7.208 | 7.222 | 7.184 | 7.246 | 7.219 | 7.222 | 7.266 | 7.23 | 7.243 | 7.219 | 7.255 | 7.217 | 7.146 | 7.147 | 7.145 | 7.145 |
| 3-Aminoisobutanoic acid | 104.071 | 0.6049 | 3.37778633 | 0.012273 | 7.132 | 7.046 | 7.184 | 7.159 | 7.2 | 6.961 | 7.286 | 7.318 | 7.49 | 7.223 | 7.22 | 7.06 | 7.087 | 7.098 | 7.095 | 7.098 |
| 5-(Hydroxymethyl)-4-methoxy-2(5H)-furanone | 145.0494 | 0.4052 | -1.197114045 | 0.013191 | 7.121 | 7.169 | 7.087 | 7.176 | 7.156 | 7.151 | 7.192 | 7.173 | 7.189 | 7.143 | 7.198 | 7.152 | 7.082 | 7.092 | 7.081 | 7.08 |
| (R)-(+)-2-Pyrrolidone-5-carboxylic acid | 130.0498 | 3.2035 | -0.463487729 | 0.004525 | 7.118 | 6.671 | 7.416 | 6.946 | 7.201 | 7.136 | 7.103 | 7.198 | 7.139 | 6.258 | 7.007 | 6.383 | 6.895 | 6.896 | 6.898 | 6.899 |
| Beta-D-Fructofuranosyl-(2->1)-beta-D-fructofuranosyl-(2->6)-alpha-D-glucopyranosyl-(1->2)-beta-D-fructofuranosyl beta-D-fructofuranoside | 811.2709 | 0.6656 | -0.57591341 | 0.003658 | 7.104 | 7.042 | 7.043 | 7.427 | 7.121 | 7.218 | 7.468 | 7.504 | 7.175 | 7.294 | 7.399 | 7.443 | 7.202 | 7.2 | 7.202 | 7.199 |
| Metazin | 391.2295 | 5.657 | -1.294255795 | 0.006376 | 7.089 | 7.141 | 7.078 | 7.122 | 7.104 | 7.112 | 7.156 | 7.137 | 7.126 | 7.093 | 7.142 | 7.103 | 7.022 | 7.018 | 7.015 | 7.016 |
| Lubiprostone | 391.2294 | 7.2571 | 1.003162366 | 0.009309 | 7.076 | 7.099 | 7.053 | 7.112 | 7.083 | 7.08 | 7.127 | 7.108 | 7.116 | 7.081 | 7.113 | 7.079 | 7.023 | 7.023 | 7.022 | 7.015 |
| DG(18:1(9Z)/18:2(9Z,12Z)/0:0) | 641.5108 | 6.796 | -1.254584769 | 0.023234 | 7.058 | 6.748 | 7.035 | 7.052 | 7.076 | 7.048 | 7.114 | 6.789 | 7.051 | 7.043 | 7.123 | 7.09 | 6.733 | 6.714 | 6.715 | 6.731 |
| 3-Hydroxychavicol 1-[rhamnosyl-(1->6)-glucoside] | 423.1604 | 0.976 | -9.841416242 | 0.008854 | 7.053 | 7.08 | 7.093 | 6.696 | 6.643 | 6.623 | 6.63 | 6.733 | 6.678 | 6.558 | 6.678 | 6.615 | 6.825 | 6.823 | 6.82 | 6.817 |
| 4-Hydroxy-L-glutamic acid | 146.0446 | 0.7042 | -0.99714586 | 0.003325 | 7.044 | 7.133 | 6.969 | 6.864 | 6.782 | 6.848 | 6.862 | 6.902 | 6.826 | 6.688 | 6.88 | 6.816 | 6.953 | 6.952 | 6.954 | 6.955 |
| 2-Hydroxycinnamic acid | 182.081 | 1.7008 | -1.082339954 | 0.00589 | 7.024 | 6.402 | 7.415 | 7.024 | 7.282 | 7.061 | 7.078 | 6.945 | 7.289 | 7.853 | 6.91 | 7.576 | 7.067 | 7.07 | 7.072 | 7.073 |
| Fagopyritol B3 | 705.185 | 0.6811 | -0.002544749 | 0.012904 | 7.02 | 7.172 | 6.594 | 6.851 | 6.919 | 6.848 | 7.149 | 7.225 | 6.9 | 6.66 | 7.138 | 6.978 | 6.844 | 6.842 | 6.854 | 6.845 |
| Hexahydro-6,7-dihydroxy-5-(hydroxymethyl)-3-(2-hydroxyphenyl)-2H-pyrano[2,3-d]oxazol-2-one | 339.1186 | 3.2035 | -0.17534534 | 0.010916 | 7.003 | 7.041 | 6.995 | 6.798 | 6.583 | 6.673 | 6.877 | 7.084 | 6.924 | 6.645 | 6.904 | 6.722 | 6.832 | 6.83 | 6.829 | 6.821 |
| Descyclopropyl Abacavir | 247.1286 | 1.9582 | -6.521635715 | 0.006666 | 6.993 | 6.716 | 6.977 | 6.297 | 6.818 | 6.666 | 6.375 | 6.604 | 6.564 | 6.1 | 6.125 | 6.158 | 6.783 | 6.778 | 6.783 | 6.778 |
| Tilarginine | 189.1344 | 0.5973 | -1.215013714 | 0.005514 | 6.947 | 6.912 | 7.164 | 6.951 | 6.889 | 6.987 | 7.096 | 7.059 | 7.028 | 6.958 | 7.038 | 6.944 | 6.751 | 6.749 | 6.75 | 6.754 |
| Chenodeoxycholylarginine | 590.4257 | 6.0819 | -3.409716245 | 0.010238 | 6.939 | 6.962 | 6.957 | 6.941 | 6.993 | 6.962 | 7.014 | 6.999 | 6.952 | 6.954 | 6.974 | 6.941 | 6.924 | 6.92 | 6.916 | 6.914 |
| (S)-Succinyldihydrolipoamide | 330.0816 | 1.6464 | 3.763916054 | 0.004887 | 6.912 | 7.171 | 6.781 | 7.004 | 7.129 | 7.061 | 6.84 | 6.845 | 6.901 | 6.556 | 6.54 | 6.584 | 7.197 | 7.193 | 7.194 | 7.191 |
| Furfural | 97.0288 | 0.6811 | 3.872459146 | 0.007602 | 6.905 | 6.85 | 6.805 | 7.027 | 6.929 | 6.972 | 7.03 | 7.018 | 6.925 | 7.001 | 6.986 | 7.042 | 6.765 | 6.762 | 6.77 | 6.765 |
| Phenylalanylasparagine | 622.2548 | 0.9293 | -8.585666679 | 0.020384 | 6.876 | 6.258 | 6.613 | 6.585 | 6.854 | 6.475 | 6.737 | 6.744 | 7.048 | 7.444 | 6.62 | 7.026 | 6.241 | 6.239 | 6.235 | 6.221 |
| Gamma-Linolenic Acid | 296.258 | 6.0663 | -1.350425776 | 0.005277 | 6.872 | 6.888 | 6.815 | 6.961 | 7.122 | 6.776 | 6.911 | 7.034 | 7.034 | 6.905 | 7.13 | 6.947 | 6.956 | 6.951 | 6.953 | 6.952 |
| Salbutamol | 520.3394 | 6.6423 | 2.65446855 | 0.012389 | 6.87 | 5.868 | 7.255 | 6.623 | 7.318 | 7.204 | 6.267 | 5.653 | 6.717 | 6.607 | 6.433 | 6.102 | 6.713 | 6.703 | 6.702 | 6.711 |
| Delmadinone | 784.3074 | 0.735 | -9.403820232 | 0.016462 | 6.848 | 6.394 | 6.771 | 6.801 | 6.842 | 6.611 | 6.905 | 6.952 | 6.928 | 7.296 | 6.8 | 7.049 | 6.455 | 6.439 | 6.443 | 6.448 |
| Dodemorph | 563.5504 | 6.2669 | -0.998025521 | 0.050497 | 6.84 | 6.847 | 6.831 | 6.902 | 6.9 | 6.773 | 6.891 | 6.872 | 6.856 | 6.865 | 6.982 | 6.833 | 6.857 | 6.873 | 6.852 | 6.82 |
| Lactose | 360.1496 | 0.6732 | -1.295893933 | 0.018043 | 6.829 | 6.716 | 6.705 | 6.909 | 6.991 | 6.867 | 6.918 | 6.85 | 7.04 | 7.083 | 6.895 | 6.987 | 6.802 | 6.821 | 6.812 | 6.81 |
| DG(18:0/18:2(9Z,12Z)/0:0) | 638.571 | 7.0723 | -1.307719669 | 0.04883 | 6.828 | 6.148 | 6.827 | 6.883 | 6.79 | 6.787 | 6.871 | 6.045 | 6.89 | 6.815 | 6.903 | 6.835 | 5.987 | 5.984 | 5.984 | 6.026 |
| 11,17-Dihydroxy-3,20-dioxopregn-4-en-21-yl acetate | 446.2532 | 5.9346 | -1.279175129 | 0.026018 | 6.814 | 6.766 | 6.665 | 6.848 | 6.784 | 6.861 | 6.867 | 6.818 | 6.797 | 6.773 | 6.864 | 6.808 | 6.642 | 6.666 | 6.666 | 6.66 |
| 15-Methylpalmitate | 288.2893 | 5.7646 | -1.6017972 | 0.009438 | 6.813 | 6.909 | 6.74 | 6.754 | 6.77 | 6.774 | 6.818 | 6.815 | 6.873 | 6.795 | 6.839 | 6.754 | 6.832 | 6.822 | 6.829 | 6.829 |
| Xi-10-Hydroxyoctadecanoic acid | 318.2998 | 5.7419 | -1.425962681 | 0.005819 | 6.812 | 6.845 | 6.764 | 6.84 | 6.817 | 6.837 | 6.84 | 6.844 | 6.89 | 6.781 | 6.813 | 6.797 | 6.731 | 6.731 | 6.727 | 6.727 |
| Dihydro-5-pentyl-2(3H)-furanone | 139.1116 | 6.4969 | -1.104387497 | 0.007842 | 6.777 | 6.711 | 6.755 | 6.824 | 6.795 | 6.792 | 6.851 | 6.752 | 6.827 | 6.816 | 6.895 | 6.839 | 6.672 | 6.669 | 6.673 | 6.677 |
| LysoPA(0:0/18:2(9Z,12Z)) | 457.2321 | 7.0264 | -0.962938895 | 0.029033 | 6.739 | 6.772 | 6.817 | 6.862 | 6.46 | 6.709 | 6.868 | 6.691 | 6.677 | 6.773 | 6.82 | 6.766 | 6.67 | 6.653 | 6.644 | 6.669 |
| Citrulline | 158.0922 | 0.5751 | -0.89009348 | 0.006546 | 6.715 | 6.907 | 6.976 | 6.919 | 6.878 | 6.991 | 6.653 | 6.51 | 6.66 | 6.805 | 6.706 | 6.702 | 6.523 | 6.521 | 6.527 | 6.524 |
| 4-Nitrophenyl sulfate | 218.9834 | 0.483 | -1.619208568 | 0.012128 | 6.71 | 7.374 | 6.792 | 7.18 | 7.104 | 7.314 | 7.257 | 7.366 | 7.089 | 7.105 | 7.221 | 7.23 | 7.105 | 7.095 | 7.105 | 7.106 |
| Isonicotinic acid | 124.0393 | 0.7042 | 0.026084693 | 0.007857 | 6.706 | 6.668 | 6.708 | 6.705 | 6.744 | 6.663 | 6.983 | 6.971 | 7.001 | 6.948 | 6.879 | 6.912 | 6.808 | 6.811 | 6.815 | 6.808 |
| Delartine | 683.3507 | 5.6802 | -4.633337042 | 0.02359 | 6.699 | 6.949 | 6.114 | 5.723 | 5.116 | 4.753 | 4.967 | 5.23 | 5.339 | 5.088 | 5.521 | 5.091 | 8.141 | 8.162 | 8.161 | 8.162 |
| Piperidine | 86.0969 | 1.7552 | 5.565735477 | 0.010861 | 6.697 | 6.259 | 6.925 | 6.575 | 6.773 | 6.525 | 6.942 | 6.878 | 7.087 | 7.394 | 6.753 | 7.167 | 6.805 | 6.803 | 6.806 | 6.814 |
| L-Proline | 116.0707 | 0.6732 | 1.038653402 | 0.007062 | 6.692 | 6.747 | 6.772 | 6.914 | 7.143 | 6.824 | 7.024 | 7.232 | 7.582 | 7.147 | 6.906 | 6.754 | 7.13 | 7.136 | 7.136 | 7.136 |
| 1-(3-phenylcinnoline-4-carbonyl)-4-piperidin-1-ylpiperidine-4-carboxamide | 444.2435 | 6.0355 | 9.185272685 | 0.016996 | 6.687 | 6.699 | 6.758 | 6.696 | 6.769 | 6.734 | 6.748 | 6.725 | 6.745 | 6.673 | 6.729 | 6.689 | 6.648 | 6.636 | 6.633 | 6.634 |
| Folcidin | 424.138 | 1.2488 | 3.551845692 | 0.006208 | 6.676 | 6.983 | 7.117 | 7.272 | 6.731 | 6.851 | 5.86 | 6.662 | 5.657 | 5.789 | 6.105 | 5.974 | 6.863 | 6.856 | 6.86 | 6.86 |
| (6S)-Tetrahydro-L-biopterin | 283.1509 | 6.6423 | -1.730006315 | 0.010027 | 6.672 | 6.688 | 6.645 | 6.717 | 6.683 | 6.687 | 6.738 | 6.686 | 6.717 | 6.692 | 6.711 | 6.696 | 6.619 | 6.617 | 6.627 | 6.62 |
| Cholylasparagine | 561.2978 | 0.5676 | 7.897760396 | 0.00945 | 6.668 | 6.775 | 6.585 | 6.795 | 6.784 | 6.814 | 6.624 | 6.27 | 6.595 | 6.745 | 6.647 | 6.711 | 6.403 | 6.403 | 6.395 | 6.397 |
| LysoPE(16:0/0:0) | 454.2924 | 6.2746 | -1.015114262 | 0.028782 | 6.659 | 5.801 | 6.869 | 6.412 | 6.712 | 6.741 | 6.098 | 5.65 | 6.462 | 6.382 | 6.403 | 6.017 | 6.405 | 6.383 | 6.382 | 6.404 |
| Oryzalexin D | 337.2733 | 6.1129 | -1.3970035 | 0.092826 | 6.655 | 6.503 | 6.702 | 6.827 | 6.805 | 6.76 | 6.767 | 6.516 | 6.668 | 6.72 | 6.745 | 6.703 | 6.55 | 6.621 | 6.587 | 6.531 |
| Apiin | 565.1548 | 3.8305 | -0.641099555 | 0.007764 | 6.651 | 6.547 | 6.635 | 7.081 | 6.512 | 6.653 | 6.673 | 7.171 | 6.545 | 6.931 | 6.777 | 7.385 | 7.811 | 7.803 | 7.808 | 7.808 |
| Verbascotetrose | 689.2105 | 0.8128 | -0.85059028 | 0.020403 | 6.641 | 6.698 | 6.561 | 6.832 | 6.705 | 6.764 | 6.785 | 6.674 | 6.56 | 6.52 | 6.792 | 6.643 | 6.363 | 6.381 | 6.363 | 6.367 |
| 3-[[(2S)-2,4-Dihydroxy-3,3-dimethylbutanoyl]amino]propanoic acid | 220.1177 | 2.2529 | -1.05509423 | 0.004527 | 6.638 | 6.413 | 6.563 | 6.523 | 6.559 | 6.518 | 6.58 | 6.535 | 6.663 | 6.459 | 6.498 | 6.366 | 6.479 | 6.481 | 6.476 | 6.478 |
| Octadecanamide | 284.2943 | 6.3665 | -1.650300382 | 0.052855 | 6.637 | 6.604 | 6.583 | 6.799 | 6.741 | 6.598 | 6.805 | 6.663 | 6.74 | 6.7 | 6.899 | 6.684 | 6.551 | 6.549 | 6.5 | 6.54 |
| L-Threonine | 120.0656 | 0.612 | 0.525451358 | 0.00521 | 6.634 | 6.44 | 6.284 | 6.381 | 6.535 | 6.246 | 6.418 | 6.314 | 6.714 | 6.838 | 6.414 | 6.517 | 6.157 | 6.159 | 6.16 | 6.162 |
| Prednisone acetate | 418.2217 | 5.888 | -1.85414576 | 0.029588 | 6.633 | 6.711 | 6.678 | 6.777 | 6.802 | 6.739 | 6.756 | 6.765 | 6.718 | 6.761 | 6.816 | 6.768 | 6.537 | 6.534 | 6.534 | 6.56 |
| CC12C(CC(CC1)C2(C)C)=CC(O)N | 228.1956 | 5.8649 | -1.216370594 | 0.007578 | 6.632 | 6.725 | 6.739 | 6.762 | 6.755 | 6.765 | 6.797 | 6.768 | 6.759 | 6.75 | 6.783 | 6.749 | 6.608 | 6.604 | 6.604 | 6.611 |
| N,N-Dimethylarginine | 203.1501 | 0.6579 | -0.67651955 | 0.008804 | 6.62 | 6.57 | 6.633 | 6.529 | 6.597 | 6.396 | 6.967 | 6.917 | 7.146 | 7.046 | 6.914 | 6.882 | 6.501 | 6.499 | 6.508 | 6.503 |
| N2-(3-Carboxy-2-hydroxy-1-oxopropyl)arginine | 291.1295 | 1.0228 | -1.427744055 | 0.017669 | 6.604 | 6.678 | 6.595 | 6.721 | 7.037 | 6.621 | 6.693 | 6.609 | 6.764 | 6.636 | 6.609 | 6.671 | 6.411 | 6.415 | 6.41 | 6.397 |
| LysoPC(20:5(5Z,8Z,11Z,14Z,17Z)/0:0) | 542.3213 | 6.7192 | -5.177643719 | 0.155502 | 6.591 | 3.787 | 6.917 | 6.126 | 6.948 | 6.779 | 5.782 | 3.859 | 6.31 | 6.178 | 6.093 | 5.58 | 5.767 | 5.622 | 5.703 | 5.774 |
| Sinistrin | 846.3078 | 1.1708 | -0.901480806 | 0.013076 | 6.586 | 6.561 | 6.516 | 6.86 | 6.59 | 6.749 | 6.938 | 6.903 | 6.789 | 6.879 | 6.844 | 6.945 | 6.609 | 6.607 | 6.619 | 6.608 |
| PE(16:0/20:2(11Z,14Z)) | 766.5306 | 6.051 | -6.856609319 | 0.006592 | 6.583 | 6.585 | 6.513 | 6.537 | 6.63 | 6.573 | 6.679 | 6.586 | 6.6 | 6.582 | 6.6 | 6.531 | 6.516 | 6.514 | 6.511 | 6.51 |
| (+/-)-1,4-Nonanediol diacetate | 245.1744 | 6.5045 | -1.282012962 | 0.009803 | 6.581 | 6.503 | 6.565 | 6.619 | 6.61 | 6.597 | 6.662 | 6.541 | 6.622 | 6.611 | 6.692 | 6.629 | 6.462 | 6.453 | 6.459 | 6.462 |
| Glucosylisomaltol | 289.0914 | 0.6811 | -1.299236592 | 0.001559 | 6.577 | 6.535 | 6.502 | 6.77 | 6.658 | 6.688 | 6.765 | 6.732 | 6.631 | 6.703 | 6.719 | 6.756 | 6.46 | 6.46 | 6.461 | 6.462 |
| Occidentoside | 353.0912 | 1.0461 | -8.941170657 | 0.013494 | 6.575 | 6.558 | 6.367 | 6.571 | 6.51 | 6.565 | 6.462 | 6.391 | 6.306 | 6.372 | 6.442 | 6.388 | 6.165 | 6.166 | 6.165 | 6.154 |
| Cinobufagin | 460.269 | 5.9346 | -0.932684022 | 0.009035 | 6.568 | 6.547 | 6.486 | 6.597 | 6.551 | 6.573 | 6.562 | 6.573 | 6.552 | 6.542 | 6.593 | 6.543 | 6.429 | 6.421 | 6.429 | 6.428 |
| Choline Phosphate | 184.0732 | 0.6049 | -3.957848012 | 0.006818 | 6.559 | 6.73 | 6.962 | 7.122 | 6.858 | 7.359 | 7.172 | 7.008 | 7.188 | 6.881 | 6.894 | 6.861 | 6.694 | 6.692 | 6.695 | 6.699 |
| L-3-Cyanoalanine | 156.0766 | 0.5526 | -1.418444879 | 0.005963 | 6.557 | 6.342 | 6.584 | 6.249 | 6.548 | 6.448 | 6.139 | 5.948 | 6.575 | 6.766 | 6.132 | 6.43 | 6.008 | 6.006 | 6.007 | 6.012 |
| 4-Propylphenol | 119.0856 | 5.9346 | 0.474646221 | 0.008979 | 6.549 | 6.571 | 6.435 | 6.624 | 6.626 | 6.586 | 6.56 | 6.64 | 6.638 | 6.598 | 6.656 | 6.574 | 6.578 | 6.581 | 6.583 | 6.588 |
| Triethanolamine | 150.1123 | 7.2955 | -1.08046276 | 0.010578 | 6.544 | 6.585 | 6.528 | 6.603 | 6.58 | 6.573 | 6.636 | 6.624 | 6.623 | 6.582 | 6.621 | 6.576 | 6.532 | 6.534 | 6.542 | 6.539 |
| Acetolein | 458.3472 | 6.1051 | -1.010921422 | 0.009638 | 6.54 | 6.474 | 6.39 | 6.417 | 6.428 | 6.432 | 6.492 | 6.476 | 6.469 | 6.443 | 6.457 | 6.462 | 6.468 | 6.458 | 6.465 | 6.463 |
| 2,6-Diaminopurine 2',3'-dideoxyriboside | 283.151 | 5.6493 | -1.452125363 | 0.011401 | 6.533 | 6.588 | 6.519 | 6.566 | 6.546 | 6.544 | 6.579 | 6.565 | 6.556 | 6.531 | 6.581 | 6.537 | 6.501 | 6.491 | 6.49 | 6.491 |
| Glycerophosphocholine | 258.1098 | 0.6198 | -1.230104321 | 0.007717 | 6.509 | 6.519 | 5.633 | 6.594 | 5.802 | 6.339 | 6.403 | 6.042 | 5.846 | 6.012 | 6.627 | 6.623 | 6.087 | 6.091 | 6.093 | 6.094 |
| (8R,9R,10S,13R,14S)-1,2,3,4,5,6,7,8,9,10,11,12,14,15,16,17-Hexadecahydrocyclopenta[a]phenanthrene-13-carbaldehyde | 278.2475 | 6.0663 | -1.321584763 | 0.008849 | 6.507 | 6.527 | 6.486 | 6.521 | 6.62 | 6.461 | 6.538 | 6.576 | 6.523 | 6.567 | 6.63 | 6.476 | 6.511 | 6.507 | 6.504 | 6.502 |
| Palmitic amide | 256.2631 | 6.2592 | -1.340964069 | 0.014519 | 6.507 | 6.538 | 6.482 | 6.625 | 6.578 | 6.49 | 6.567 | 6.571 | 6.624 | 6.546 | 6.652 | 6.526 | 6.525 | 6.513 | 6.512 | 6.513 |
| (2S,3R)-2-Amino-4-octadecene-3-ol | 316.3206 | 5.8111 | -1.384460142 | 0.010196 | 6.505 | 6.607 | 6.446 | 6.479 | 6.497 | 6.48 | 6.532 | 6.547 | 6.59 | 6.492 | 6.56 | 6.503 | 6.601 | 6.592 | 6.592 | 6.595 |
| 5-Methylthioribose | 222.0793 | 1.0306 | -0.743912092 | 0.012094 | 6.504 | 6.311 | 6.466 | 6.231 | 6.282 | 6.205 | 6.486 | 6.401 | 6.587 | 6.464 | 6.342 | 6.282 | 6.073 | 6.082 | 6.07 | 6.071 |
| Laninamivir | 391.1208 | 1.9816 | 2.413556585 | 0.011846 | 6.497 | 5.946 | 6.58 | 6.339 | 6.396 | 6.055 | 6.229 | 6.231 | 6.35 | 6.147 | 6.297 | 5.936 | 6.057 | 6.055 | 6.057 | 6.066 |
| Triglutamate folate | 867.237 | 0.6501 | 7.680094895 | 0.003026 | 6.489 | 6.63 | 5.988 | 6.378 | 6.418 | 6.3 | 6.951 | 7.058 | 6.772 | 6.529 | 6.868 | 6.802 | 6.726 | 6.725 | 6.727 | 6.724 |
| N-(2-(Diethylamino)ethyl)-5-((5-fluoro-2-oxoindolin-3-ylidene)methyl)-2,4-dimethyl-1H-pyrrole-3-carboxamide | 416.2485 | 3.4431 | 7.130185096 | 0.020561 | 6.489 | 6.538 | 6.457 | 6.514 | 6.522 | 6.503 | 6.555 | 6.532 | 6.532 | 6.479 | 6.529 | 6.496 | 6.463 | 6.445 | 6.445 | 6.446 |
| L-Aspartic acid | 134.0447 | 0.6049 | -0.755646986 | 0.006946 | 6.481 | 6.379 | 6.32 | 6.475 | 6.32 | 6.176 | 6.335 | 6.151 | 6.332 | 6.385 | 6.428 | 6.505 | 6.115 | 6.118 | 6.121 | 6.122 |
